# Role of the m^6^A methyltransferase Mettl16 in *Drosophila* development

**DOI:** 10.1371/journal.pgen.1012317

**Published:** 2026-09-24

**Authors:** Penghui Song, Lijuan Ma, Dong Yan

**Affiliations:** 1 State Key Laboratory of Genetics and Development of Complex Phenotypes, School of Life Sciences, Fudan University, Shanghai, China; 2 CAS Center for Excellence in Molecular Plant Sciences, Institute of Plant Physiology and Ecology, Chinese Academy of Sciences, Shanghai, China; University of Michigan, UNITED STATES OF AMERICA

## Abstract

N^6^-methyladenosine (m^6^A), one of the most abundant chemical modifications on RNA, is installed by METTL3 and several other methyltransferases, including METTL16. Although METTL16 has been characterized in several model organisms, its function in *Drosophila* remains unknown. Here, we show that, unlike in mammals, *Drosophila Mettl16* mutants are viable but exhibit multiple developmental and behavioral defects. Both male and female mutants are sterile, with severe gametogenesis defects. *Mettl16* mutant germ cells can pass the mitotic and meiotic stages, but are defective in spermatid elongation and individualization. Mettl16-GFP localizes predominantly to the nucleus, and its absence does not impair global protein synthesis in wing disc epithelial cells. MeRIP-Seq analysis indicates that Mettl16 loss affects m^6^A on only a small subset of transcripts, in contrast to the broad effect of Mettl3. We further demonstrate that Mettl16 interacts with U6 snRNA and is required for its m^6^A modification. Accordingly, *Mettl16* mutants display widespread alterations in alternative splicing. Together, the pleiotropic phenotypes of *Mettl16* mutants likely stem from its role in m^6^A deposition and U6-dependent splicing regulation of specific target transcripts.

## Author summary

RNA molecules carry chemical modifications that act as key regulators of their function. Among the most common is m^6^A, which is added by several “writer” proteins. One of these writers, Mettl16, is conserved across species, yet its in vivo role remains less understood, especially in the fruit fly *Drosophila*. In this study, we uncover the key functions of Mettl16 in fly development and reproduction. Flies lacking Mettl16 are viable but show a range of developmental and behavioral problems. Interestingly, both males and females are sterile, with severe defects in sperm and egg production. Mechanistically, Mettl16 only methylates a small subset of mRNAs, unlike the broad activity of the major writer Mettl3. Loss of Mettl16 reduces the m⁶A modification on U6 snRNA, a core component of the splicing machinery, and affects alternative splicing on a wide scale. We propose that the diverse defects in *Mettl16* mutants arise from its role in installing m⁶A on specific transcripts and in ensuring accurate splicing through U6 modification.

## Introduction

Epitranscriptomics, which encompasses over 170 chemical modifications on mRNA and non-coding RNAs (ncRNAs), represents a major regulatory layer in gene expression [1,2]. Among these modifications, N^6^-methyladenosine (m^6^A) is the most prevalent internal mark on eukaryotic mRNA and has driven much of the field’s progress [3,4], aided by continually evolving transcriptome-wide mapping technologies [5,6]. Biochemical and genetic studies have characterized the core m^6^A machinery comprising methyltransferases (“writers”), demethylases (“erasers”), and m^6^A-binding proteins (“readers”) [7,8]. The writer complex includes the METTL3-METTL14 catalytic core (MAC) and associated regulatory subunits such as WTAP, VIRMA, RBM15, ZC3H13, and HAKAI (collectively termed MACOM). The erasers FTO and ALKBH5 reverse the modification, while readers, predominantly the YTH domain-containing proteins YTHDC1–2 and YTHDF1–3, interpret the m^6^A signal. Over the past decade, m^6^A has been implicated in a broad range of biological processes, including development, stem cell regulation, immune responses, memory formation, and tumorigenesis [9], leading to impactful translational discoveries [10].

Although METTL3 deposits m^6^A on thousands of transcripts, other enzymes also contribute to the m^6^A landscape [11]. For instance, PCIF1 generates m^6^Am when the first transcribed, 2’-O-methylated nucleotide of an mRNA is adenosine [12–15]. Furthermore, METTL5 and ZCCHC4 catalyze single m^6^A modifications on 18S and 28S ribosomal RNA (rRNA), respectively [16–21]. Within the spliceosome, the small nuclear RNAs (snRNAs) U2 and U6 are modified by METTL4 (producing m^6^Am) and METTL16 (producing m^6^A), respectively [22–26]. Interestingly, while a METTL3 homolog is absent in some organisms (e.g., nematodes), METTL16 is evolutionarily conserved from bacteria and yeast to vertebrates [23]. All METTL16 homologs share a methyltransferase domain, while vertebrate versions contain two additional vertebrate-conserved regions (VCRs). Unlike METTL3, which methylates single-stranded RNA bearing the consensus “RR**m**^**6**^**A**CH” motif, METTL16 primarily modifies structured RNA hairpins containing the “UAC**m**^**6**^**A**GARAA” (R = A or G) sequence.

Crosslinking and analysis of cDNA (CRAC) experiments have shown that METTL16 binds not only to U6 snRNA but also to other ncRNAs, pre-mRNAs, and lncRNAs such as MALAT1 [22]. In human cells, METTL16 installs m^6^A at position A43 of U6 snRNA and interacts with U6 biogenesis factors including La, LARP7, and the methylphosphate capping enzyme MEPCE. Conservation of this function has been demonstrated in *S. pombe*, *A. thaliana*, and *C. elegans*, where METTL16 homologs similarly modify U6 [23,27–29]. Mechanistically, m⁶A within the U6 “ACAGA” box base-pairs with intronic sequences downstream of the 5′ splice site during splicing; loss of METTL16 therefore causes widespread splicing defects, particularly at 5′ splice sites [22,30].

Another key target of METTL16 is the mRNA encoding S-adenosylmethionine (SAM) synthetase (MAT2A). SAM synthetase produces SAM, the universal methyl donor for cellular methylation reactions. In human cells, METTL16 methylates a series of hairpin structures in the 3′UTR of *MAT2A* mRNA, thereby regulating its stability and splicing [23,31,32]. In *C. elegans*, the METTL16 ortholog METT-10 deposits an m^6^A mark at the 3’ splice site of the SAM synthetase pre-mRNA, inhibiting splicing and reducing protein output [29]. This METTL16-dependent regulation of SAM synthetase is nutrient-responsive, helping maintain cellular SAM homeostasis. Besides its nuclear roles, METTL16 has also been detected in the cytoplasm, where it interacts with eukaryotic initiation factors (eIFs) and promotes translation in an m^6^A-independent manner [33].

*Drosophila* has served as a powerful model for studying RNA modifications [34–36]. Its well-defined sex-determination system enabled the identification of several m^6^A MACOM components and clearly established a role for m^6^A in splicing regulation [37–43]. Although METTL16 function has been explored in multiple model organisms, its biological and molecular roles in *Drosophila* remain uncharacterized. In this study, we generate multiple genetic tools to define the role of Mettl16 during fly development and investigate its molecular mechanisms. Our findings provide important insights into Mettl16-dependnent regulation of developmental processes.

## Results

### *Mettl16* mutants exhibit multiple developmental and behavioral phenotypes

**Fig 1:**
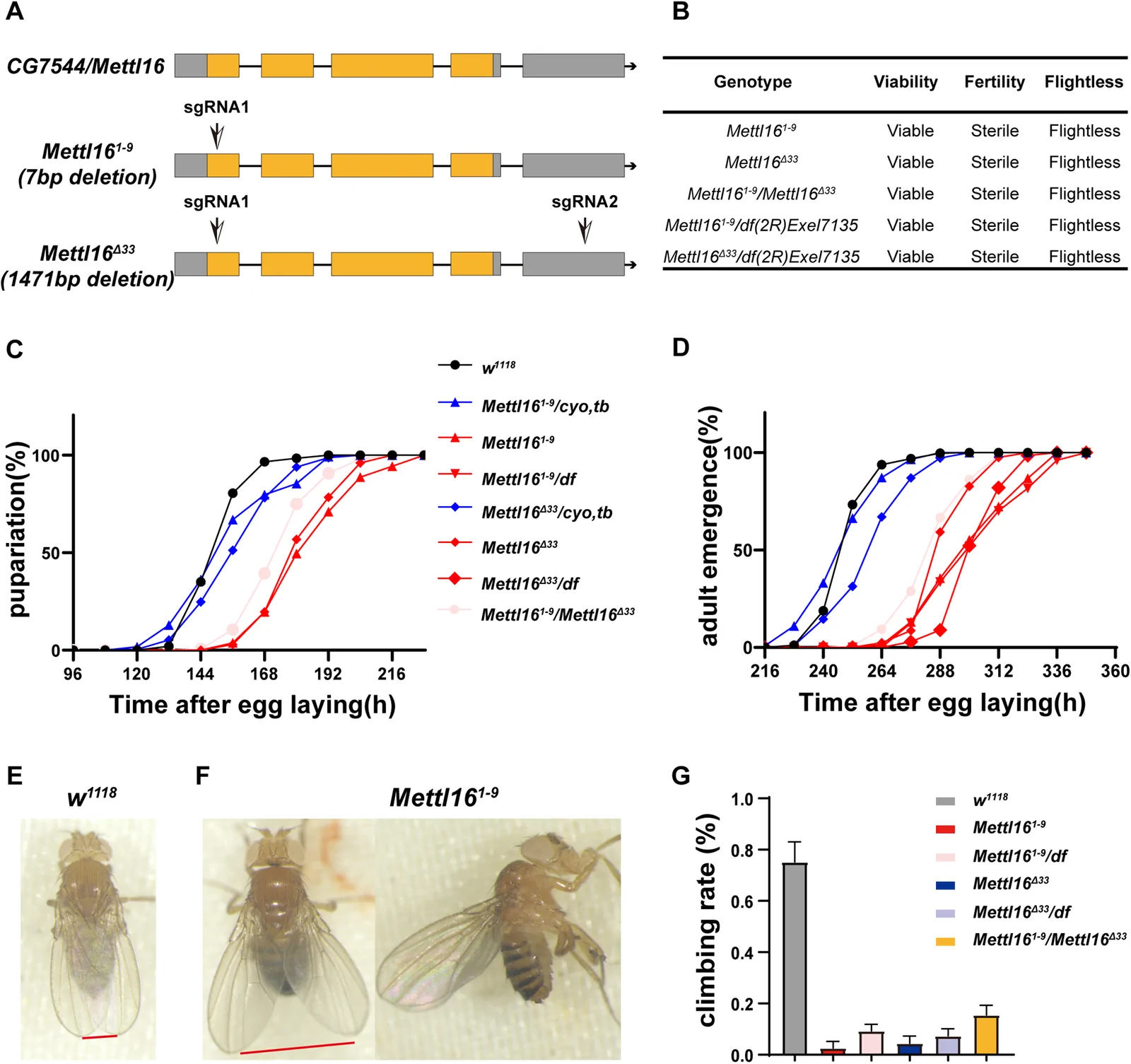
Phenotypic analysis of *Mettl16* mutants. **(A)** Schematic diagrams of the *Mettl16* alleles. Exons are represented by boxes, with gray and yellow indicating non-coding and coding regions, respectively. The sgRNA-targeting sites (arrows) and the resulting sequence changes are indicated. **(B)** Summary of viability, fertility, and flightless phenotypes for the indicated combinations of *Mettl16* alleles and a deficiency covering *Mettl16* locus. **(C-D)** Pupariation time (C) and adult eclosion time (D) for flies of the indicated genotypes. **(E-F)** Wing posture of control *w*^*1118*^ (E) and *Mettl16*^*1*^*^–^*^*9*^ mutant (F) adult flies. **(G)** Climbing assay. The percentage of flies (n > 45 per genotype) that climbed to a height of 5 cm within a fixed time period was quantified.

*Drosophila* has an ortholog of mammalian *METTL16*, designated *CG7544*, which is located on chromosome 2R and encodes a 305aa protein (hereafter referred to as *Mettl16*). To elucidate its biological functions, we first used CRISPR/Cas9 technology to generate a 7-base-pair (bp) deletion in exon 1 of *Mettl16* by sgRNA1 (Fig 1A). This frameshift allele leads to a premature stop codon and was designated *Mettl16¹^–^⁹*. Notably, unlike previously reported mammalian *METTL16* mutants that show early embryonic lethality, *Mettl16¹^–^⁹* homozygous flies are viable. To rule out the possibility of residual Mettl16 activity in the *Mettl16¹^–^⁹* homozygotes, we created a second allele, *Mettl16*^*Δ*^*³³*, with a 1471-bp deletion generated by combining sgRNA1 and sgRNA2 (the later targeting the 3’ UTR of *Mettl16*) (Fig 1A). This large deletion removes most of the *Mettl16* coding sequencing and is thus considered a putative null allele.

We confirmed by qPCR that both mutants show significantly reduced levels of *Mettl16* mRNA (S1A Fig). Using a custom polyclonal antibody generated against full-length Mettl16 protein, we detected a band of the expected size in control flies via Western blot. Although this antibody recognizes several non-specific bands, the band corresponding to Mettl16 was absent in both mutants (S1B Fig). Furthermore, we obtained a deficiency, *Df(2R)Exel7135*, covering the *Mettl16* locus. Homozygous mutants, trans-heterozygotes of the two alleles, and heterozygotes over the deficiency all exhibit identical phenotypes (Fig 1B; see below), indicating that both *Mettl16* alleles are likely functional nulls.

Although viable, *Mettl16* mutants exhibit several distinct phenotypes. First, both pupariation and eclosion were delayed by approximately 24–48 hours in various *Mettl16* mutants (red lines) compared to controls (black and blue lines) (Fig 1C and 1D). Second, while *w¹¹¹⁸* control flies typically keep their wings in a folded position (Fig 1E), most *Mettl16¹^–^⁹* mutants fail to fold their wings properly and display held-out wings (Fig 1F, left). In addition, some of these flies have their wings raised upward (Fig 1F, right). Third, *Mettl16* mutants are completely flightless (Fig 1B) and show severely impaired climbing ability (Fig 1G).

### Mettl16 is a ubiquitously expressed nuclear protein

**Fig 2:**
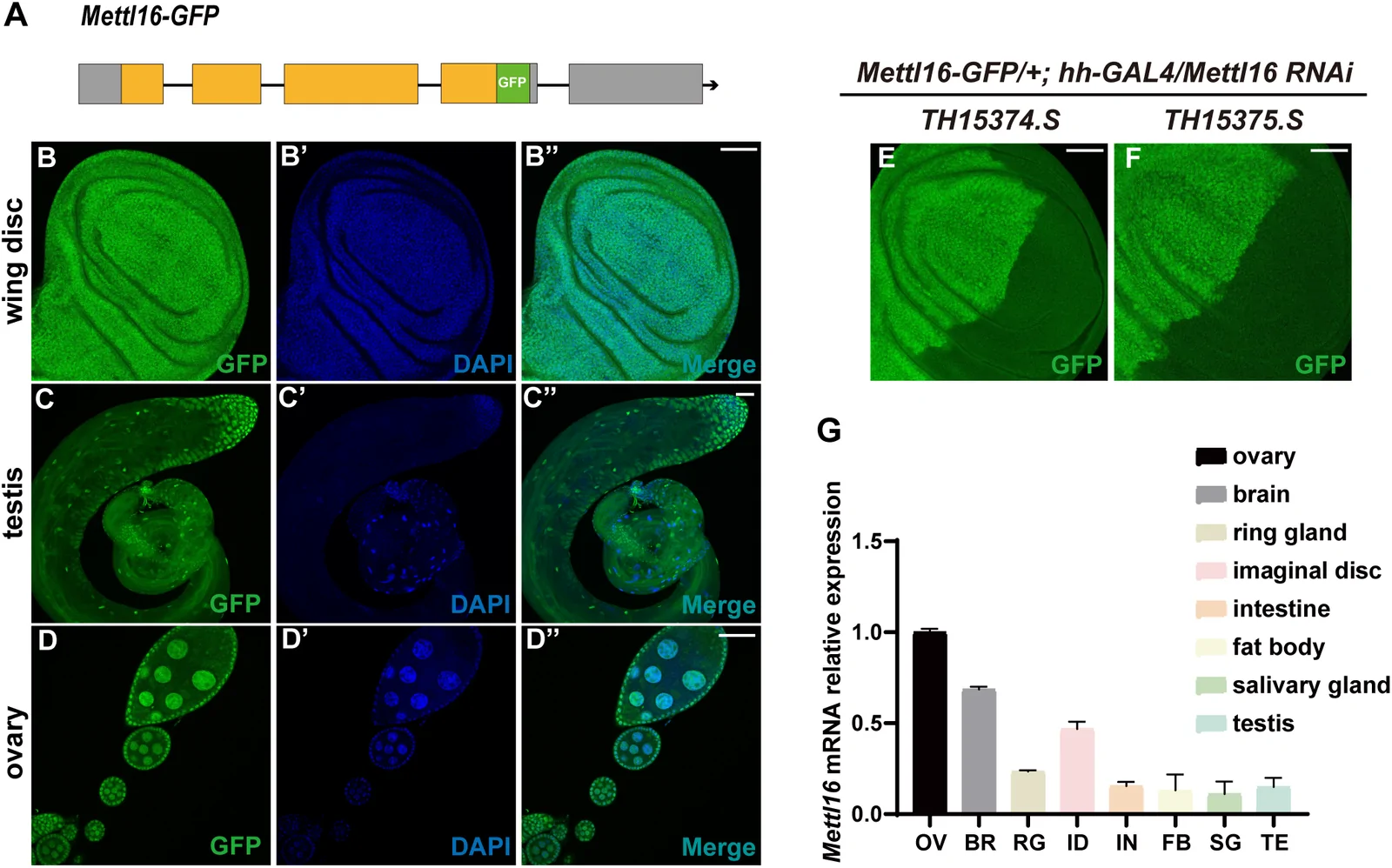
Mettl16 is a widely expressed nuclear protein. **(A)** Schematic of the *Mettl16-GFP* knock-in fly. (B-D”) Wing discs (B-B”), testis (C-C”), and ovaries (D-D”) from *Mettl16-GFP* flies stained with anti-GFP (green) and DAPI (blue), showing nuclear co-localization. **(E-F)** Depletion of Mettl16-GFP signal in the posterior region upon expression of two independent *Mettl16* RNAi lines driven by *hh-GAL4*. Scale bars: 50 μm. **(G)** Relative expression levels of *Mettl16* mRNA across different tissues, as measured by qPCR.

We next examined the expression profiles of Mettl16 mRNA and protein. qPCR results show that *Mettl16* is expressed in multiple tissues including ovary, brain, ring gland, imaginal discs, intestine, fat body, salivary gland, and testis, with highest levels in the ovary and brain (Fig 2G). Because our custom antibody was not suitable for immunostaining, we generated a knock-in *Mettl16-GFP* line that has GFP inserted into the C terminus of Mettl16 ORF (Fig 2A). In wing discs, Mettl16-GFP appears as a ubiquitously expressed nuclear protein that co-localizes well with DAPI (Fig 2B–2B”). It shows a similar pattern in germline and somatic cells of testis and ovary (Fig 2C–2D”). We further generated two shRNA lines against *Mettl16*, which result in almost complete knockdown of Mettl16-GFP when induced in the posterior region of wing discs by *hh-GAL4* (Fig 2E and 2F).

### *Mettl16* mutants are sterile in both males and females

**Fig 3:**
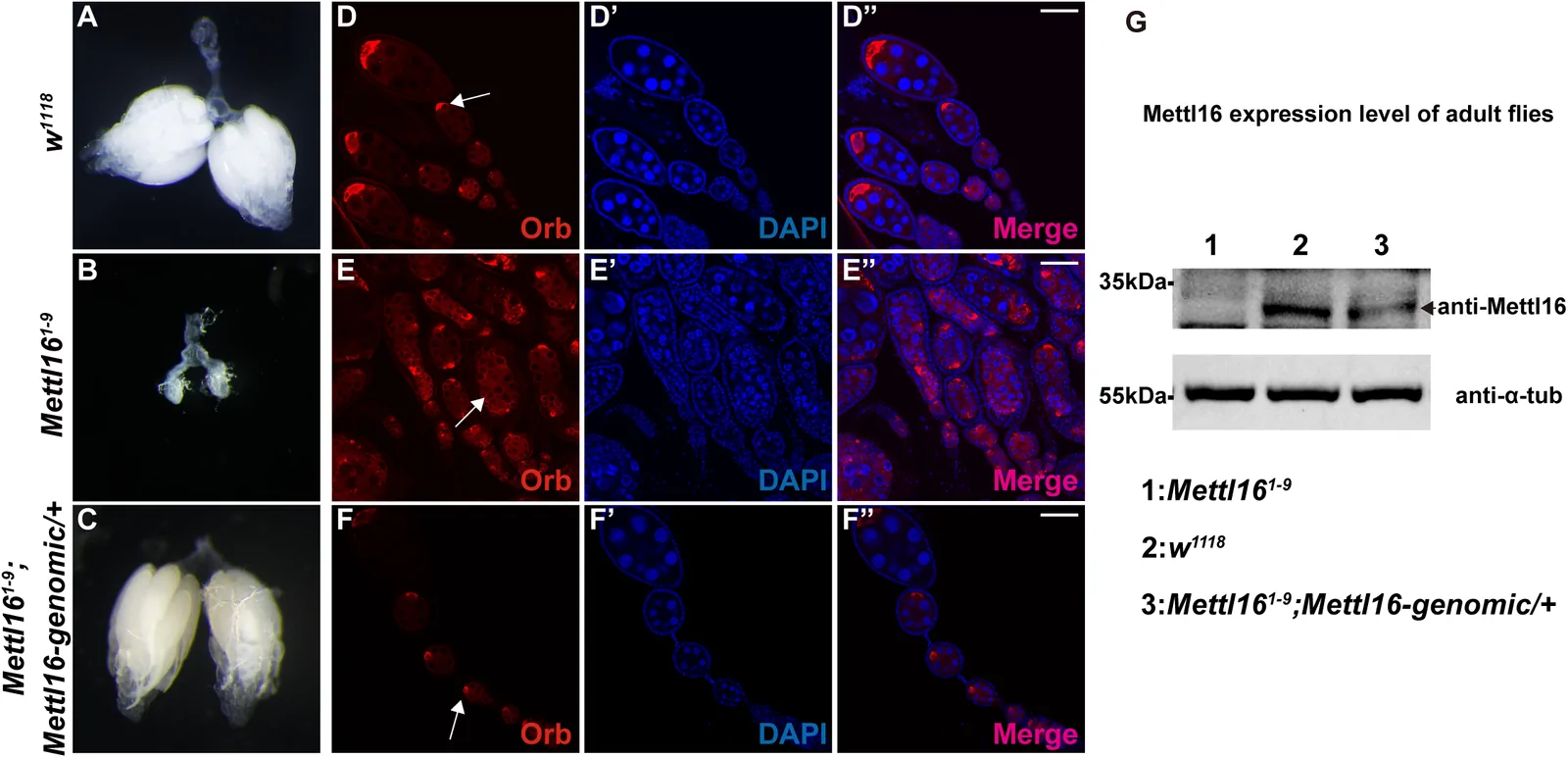
*Mettl16* is required for oogenesis. **(A-C)** Bright-field images of ovaries. (D-F”) Ovaries stained for Orb (red) and DAPI (blue). Orb is restricted to the posterior oocytes in controls (D, arrow) but is distributed diffusely in *Mettl16* mutants (E, arrow). The polarity of Orb is restored in the rescue (F, arrow) Scale bars: 50 μm. **(G)** Western blot analysis of Mettl16 protein levels in the indicated genotypes.

In addition to developmental and behavioral defects, *Mettl16*^*1*^*^–^*^*9*^ mutants are sterile in both sexes. First, we examined the female reproductive system. *Mettl16*^*1*^*^–^*^*9*^ ovaries are markedly smaller than *w¹¹¹⁸* controls and lack mature egg chambers (Fig 3A and 3B). We stained these ovaries with DAPI and an anti-Orb antibody. In control ovaries, Orb is normally restricted to the posterior region of the oocyte (Fig 3D–3D”). In contrast, *Mettl16* mutant egg chambers contain abnormal numbers of germline cells and exhibit mislocalized Orb staining (Fig 3E–3E”).

Importantly, we generated a rescue transgene containing a genomic fragment encompassing the *Mettl16* locus with its native regulatory elements. This construct restored most Mettl16 protein expression in the *Mettl16¹^–^⁹* background (Fig 3G) and fully rescued female sterility, as well as the ovary phenotypes (Fig 3C, 3F–3F”). Collectively, these results demonstrate that Mettl16 is essential for oogenesis in *Drosophila*.

We further asked which tissue requires *Mettl16* in this process using tissue-specific RNAi knockdown. When *Mettl16* RNAi was induced in germline cells by *nanos-GAL4:VP16* or in the reproductive somatic cells by *tj* (*traffic jam*)*-GAL4*, these females can still lay eggs. However, when we stained these ovaries with DAPI and Orb, they consistently contained egg chambers with abnormal numbers of germline cells and mislocalized Orb staining (S3B–S3E” Fig). Therefore, *Mettl16* knockdown in germline or reproductive somatic cells has a mild phenotype, and the sterility of the *Mettl16* mutants likely arises from combinatorial effects across germline, reproductive somatic tissues, and potentially other organs.

### *Mettl16* mutants are defective in spermatid elongation and individualization

We then analyzed the male reproductive system. While the seminal vesicles of *w¹¹¹⁸* control males contain mature sperm, those of *Mettl16*^*1*^*^–^*^*9*^ mutants are empty, consistent with their sterility (Fig 3A–3D). During spermatogenesis, a spermatogonium undergoes four mitotic divisions to produce 16 primary spermatocytes, which then complete two meiotic divisions to yield 64 spermatids. These spermatids subsequently differentiate into mature sperm via flagellar elongation and the individualization process [44]. Bright-field imaging of both control and *Mettl16* mutant testes revealed elongated flagellar structures (S2A and S2B Fig), indicating that spermatogenesis in the mutant can proceed through mitosis and meiosis to the elongation stage. Immunostaining for Vasa, a germ‑cell marker, showed 8-, 16-, and 32-cell cysts in both genotypes (S2A’ and S2B’ Fig). Moreover, staining for Histone H3 revealed no obvious defects in round spermatids after meiosis in *Mettl16* mutants compared with controls (S2C and S2D Fig).

**Fig 4:**
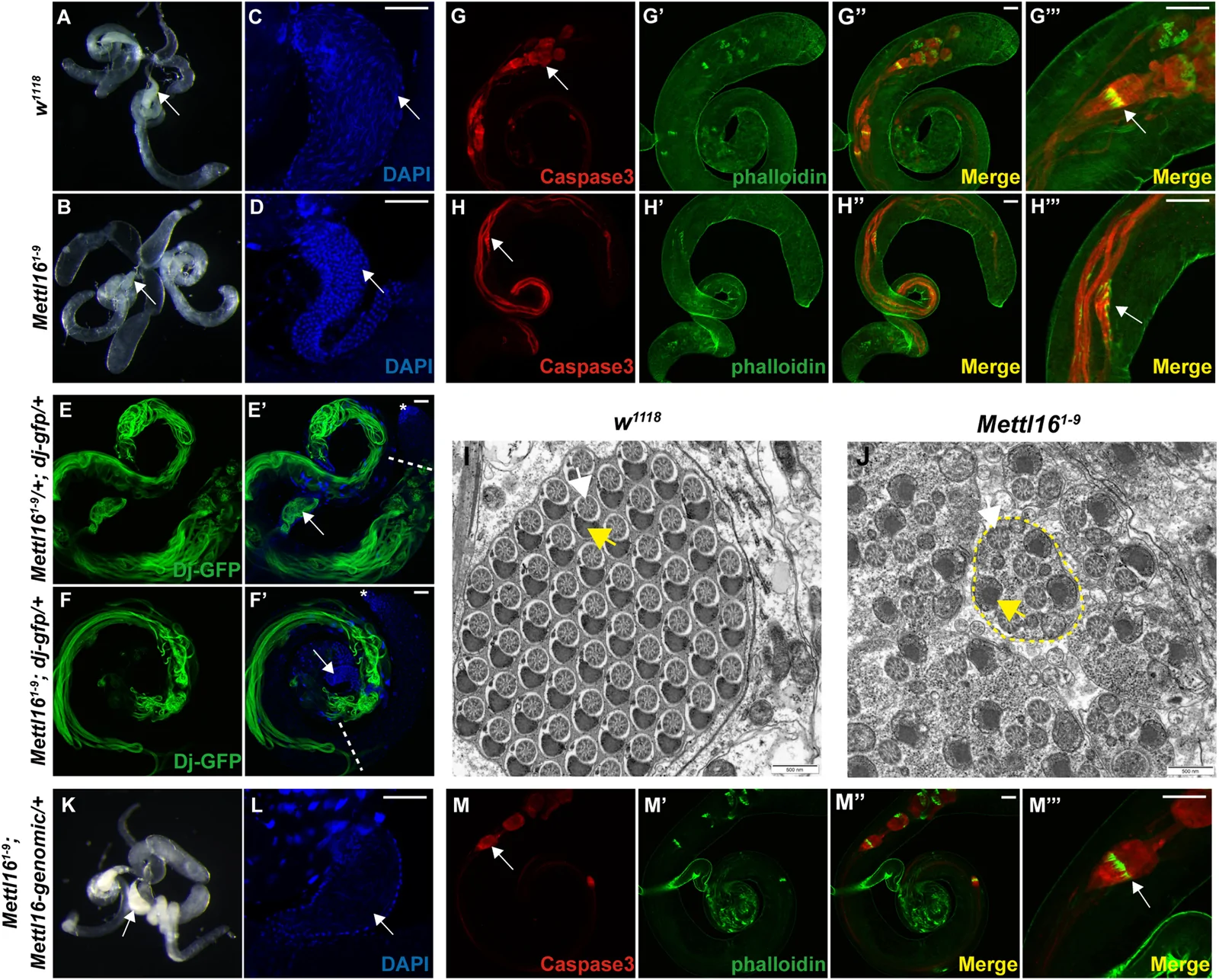
*Mettl16* mutants exhibit multiple defects in spermatogenesis. **(A-B)** Bright-field images of testes. The seminal vesicle (arrow) is filled with sperm in controls (A) but empty in *Mettl16*^*1*^*^–^*^*9*^ mutants **(B)**. **(C-D)** DAPI staining of seminal vesicles. Needle-shaped sperm nuclei are visible in controls (C, arrow) but absent in mutants **(D)**. (E-F’) Dj-GFP (green) labels elongating sperm tails. In wild-type, they fill the seminal vesicle (E’, arrow). In mutants, tails are present in the testis but the seminal vesicle is empty (F’, arrow). Control flagella extend close to the stem cell region (E’, marked with “*”), whereas mutant flagella extend only about halfway (F’, longer distance between dashed line and “*”). (G-H”’) Testes stained for cleaved Caspase-3 (red, marking waste bags) and phalloidin (green, labeling actin cones). Higher-magnification views are shown in G”’ and H”’. Scale bars: 50 μm. **(I-J)** Transmission electron micrographs of testis cross-sections. Wild-type cysts show 64 spermatids with axonemes (I, white arrow) and mitochondria derivatives (I, yellow arrow) within a common membrane. This structure is disorganized in mutants (J, arrows). (K-M”’) The defects are rescued by a genomic *Mettl16* transgene. The seminal vesicles contain sperms (K and L, arrows), and the individualization process is restored (M-M”’). Scale bars: 50 μm.

After meiosis, sperm flagella begin to elongate. We monitored this process using Don Juan-GFP (Dj-GFP), a late marker for sperm elongation. Compared with the control, most mutant flagella extended only about halfway along the testis length, and Dj-GFP signal was absent from the mutant seminal vesicle (Fig 4E–4F’). During the individualization stage, actin-rich individualization complexes (ICs) can be visualized by phalloidin staining. In control testes, ICs are neatly aligned and move toward the testis tip, expelling excess cytoplasm and organelles to form apoptotic “waste bags” that are labeled by an antibody against activated Caspase-3 (Fig 4G–4G’’’). In contrast, *Mettl16* mutants displayed disorganized ICs and a complete absence of waste bags, indicating a failure in individualization (Fig 4H–4H’’’).

We further examined sperm cysts during the individualization process by transmission electron microscopy (TEM). In control testes, individualized sperm retain only minimal residual cytoplasm, and each axoneme-mitochondrion complex is tightly encased by a distinct, intact plasma membrane (Fig 4I). In mutant samples, by contrast, only a small number of axoneme-mitochondrion complexes were present; many sperm tails remained bundled within a common membrane and displayed extensive cytoplasmic retention (Fig 4J), confirming a failure in individualization. Notably, both the sterility and the individualization defects are fully rescued by the *Mettl16* genomic transgene (Fig 4K–4M’’’).

We also examined the role of *Mettl16* in different male reproductive tissues. Mettl16-GFP is expressed in germline cells as well as in somatic cells, as indicated by co-staining with Eya (S3A–S3A’’’ Fig). Male flies in which *Mettl16* RNAi was induced by *nanos-GAL4:VP16* or *tj-GAL4* produced strongly reduced progeny numbers. Similar to the mutants, their testes showed absence of waste bags and disorganized actin cones (S3F–S3I” Fig). Consistent with the female data, Mettl16 plays a role in both germline and somatic tissues of the male reproductive system.

### Depletion of Mettl16 does not affect translation efficiency in wing discs

In human cells, METTL16 has been observed in the cytoplasm, where it interacts with eIF3a, eIF3b, and ribosomal RNAs to facilitate translation [33]. To investigate the molecular mechanism of *Drosophila* Mettl16, we first asked whether it influences protein synthesis using an O-propargyl-puromycin (OPP) incorporation assay, which serves as a readout of global translation activity [45]. In control wing imaginal discs, OPP signal was uniformly distributed (S4A–S4A” Fig). Overexpression of Rheb, an activator of the TOR pathway, in the posterior compartment by *en‑GAL4, UAS‑GFP* strongly increased OPP incorporation (S4B–S4B” Fig) [46,47]. RNAi knockdown of PPP1R15, a phosphatase in the integrative stress response, led to reduced OPP staining in the posterior compartment, likely through enhanced eIF2α phosphorylation (S4C–S4C” Fig) [48]. Importantly, induction of two independent shRNAs against *Mettl16* (previously validated in Fig 2E and 2F) in the posterior compartment did not alter OPP levels (S4D–S4E” Fig). On the other hand, we also generated a *UAS-Mettl16* transgenic line, and its overexpression had no effect on OPP signal (S4F–S4F” Fig). To corroborate the RNAi results, we performed clonal analysis using the *Mettl16* ^*1*^*^–^*^*9*^ allele and observed no change in OPP levels in control or mutant clones (S4G–S4G” Fig). These data suggest that Mettl16 does not regulate global translation in disc cells.

### Mettl16 methylates only a small subset of RNAs in *Drosophila*

**Fig 5:**
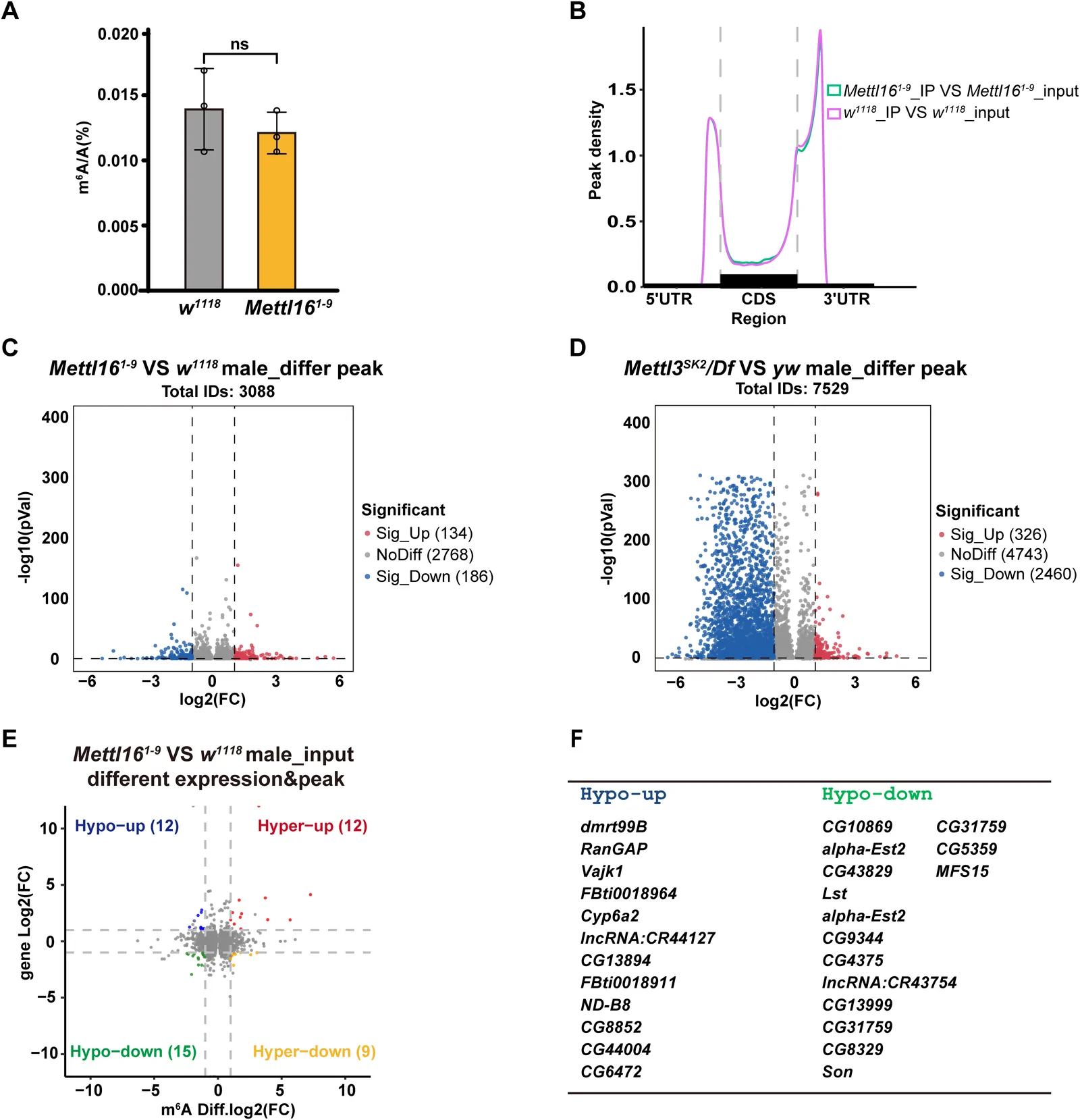
Mettl16 methylates a small subset of RNAs. **(A)** LC‑MS quantification of m⁶A/A ratios in poly(A)⁺ mRNA from adult male flies. m⁶A levels are reduced by ~10% in *Mettl16¹*^–^*⁹* mutants relative to *w¹¹¹⁸* controls. **(B)** Metagene profile of m⁶A peak density across mRNA regions (5′UTR, CDS, 3′UTR) in *Mettl16¹*^–^*⁹* and *w¹¹¹⁸* adult males. **(C, D)** Genes with differential m^6^A peaks in *Mettl16* (C) and *Mettl3* (D) mutants versus controls. Blue indicates genes with significantly decreased m^6^A peaks, and red indicates genes with upregulated peaks (P < 0.05, |log2FC| > 1). **(E)** Four-quadrant plot showing differential m^6^A peaks with differential mRNA expression in *Mettl16*^*1*^*^–^*^*9*^ mutants versus *w¹¹¹⁸*. “Hyper-” or “Hypo-” indicates increased or decreased m^6^A peak intensity; “Up” or “Down” indicates increased or decreased mRNA expression levels. Number of significantly changed genes are labeled (P < 0.05, |log2FC| > 1). **(F)** List of genes exhibiting both decreased m⁶A peaks and altered expression from **(E)**.

We next examined the role of Mettl16 as an m^6^A methyltransferase. Total RNA was isolated from *w¹¹¹⁸* control and *Mettl16*^*1*^*^–^*^*9*^ mutant adults and subjected to two rounds of poly(A) selection, followed by quantitative liquid chromatography‑mass spectrometry (LC‑MS) to measure m⁶A levels. Compared with the control, the m⁶A level in *Mettl16* mutants decreased by only about 10% (Fig 5A). These results indicate that Mettl16 is not a major m⁶A methyltransferase in *Drosophila*, which aligns with earlier reports in human cells.

To find the transcripts specifically methylated by Mettl16, we performed methylated RNA immunoprecipitation sequencing (MeRIP-seq) on *Mettl16*^*1*^*^–^*^*9*^ and *w¹¹¹⁸* male flies, with three biological replicates per genotype. A peak-calling algorithm was applied to identify m^6^A peaks (S1 Table). The peak density plot in *w¹¹¹⁸* flies showed m^6^A enrichment in the 3’ UTR near the stop codon, and to a lesser extent, in the 5’ UTR around the start codon (Fig 5B, purple line), consistent with our previous report [37]. The distribution of m^6^A peaks in *Mettl16* mutants was similar (Fig 5B, green line), again indicating only a mild global change in m^6^A modification.

We then focused on differentially methylated peaks upon m^6^A writer depletion. From our published dataset on *Mettl3* mutants, we identified 2460 significantly decreased and 326 increased m^6^A peaks compared to control (P < 0.05 and |log_2_FC| > 1) (Fig 5D) [37]. Notably, *Mettl16* mutants showed only 186 decreased and 134 increased m⁶A peaks (P < 0.05 and |log_2_FC| > 1) (Fig 5C, S2 Table). These data demonstrate that while Mettl3 acts as the major m^6^A methyltransferase in *Drosophila*, Mettl16 methylates only a small subset of mRNAs. Gene Ontology (GO) and KEGG pathway enrichment analyses revealed that the differentially methylated genes in *Mettl16* mutants are associated with neuron-related processes and in RNA-processing pathways (S5A and S5B Fig).

Next, we analyzed gene expression changes regulated by Mettl16 via RNA-Seq (S3 Table). In *Mettl16* mutants compared to control flies, 610 genes were upregulated and 809 genes were downregulated (S5C Fig, S4 Table). GO enrichment analysis revealed that these differentially expressed genes are associated with terms such as sexual reproduction, consistent with the observed sterility phenotype (S5D Fig). To identify potential direct regulatory targets of Mettl16, we integrated the RNA-Seq data with our m⁶A profiling results (Fig 5E, S5 Table). This combined analysis identified 27 genes that exhibited both decreased m^6^A peak intensity and altered expression levels: 12 were upregulated and 15 were downregulated (Fig 5F). These genes represent strong candidates for mediating the diverse biological phenotypes caused by *Mettl16* loss.

### Mettl16 does not methylate *Sams* mRNA under normal conditions

It has been reported that in mammalian cells and *C. elegans*, METTL16 methylates the 3’UTR or the 3’ splicing site of SAM synthetase mRNA, respectively, thereby regulating its expression level [23,29]. To explore a potential similar mechanism in flies, we compared m^6^A levels on *Sams* transcripts between *Mettl16*^*1*^*^–^*^*9*^ mutants and *w¹¹¹⁸* controls. First, although m^6^A peaks were detected in the 3’UTR of the *Sams* transcripts, their signal did not decrease in *Mettl16* mutants (S6A Fig, right arrow). Second, while the known Mettl16 motif UACAGAAAC is present at the junction of intron 4 and exon 5, no m^6^A peaks were identified in this region (S6A Fig, left arrow). Third, qPCR analysis showed that the levels of mature *Sams* mRNA or intron 4 were unchanged in *Mettl16* mutants (S6B Fig). Together, these results indicate that under normal culture conditions, *Drosophila* Mettl16 does not catalyze m^6^A modification of *Sams* transcripts nor regulate their expression.

### Mettl16 mediates m⁶A modification of U6 snRNA in *Drosophila*

**Fig 6:**
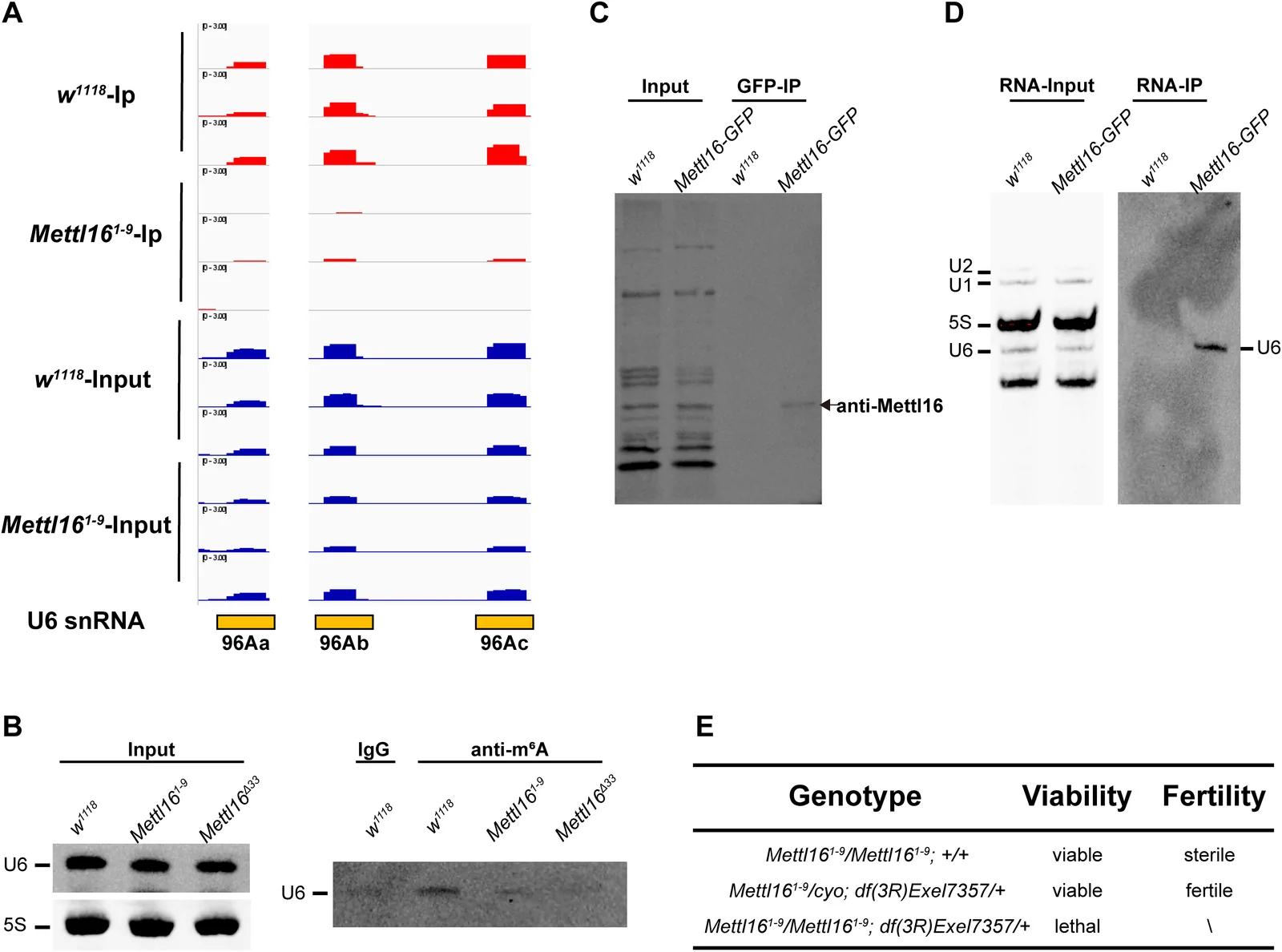
Mettl16 mediates m^6^A modification of U6 snRNA. **(A)** MeRIP-seq (red, IP) and RNA-seq (blue, input) coverage tracks for three *Drosophila* U6 snRNA genes in *Mettl16*^*1*^*^–^*^*9*^ and *w*^*1118*^ flies. m^6^A peak signals are reduced in the mutant. (B) m^6^A immunoprecipitation (m^6^A-IP) followed by Northern blot analysis. Total U6 and 5S rRNA levels (Input) are comparable across genotypes. Enrichment of U6 in the m⁶A-IP fraction is markedly reduced in *Mettl16¹*^–^*⁹* and *Mettl16*^*Δ*^*³³* mutants. **(C, D)** RNA immunoprecipitation (RIP) validates the Mettl16-U6 snRNA interaction. **(C)** Adult fly lysates were immunoprecipitated using GFP nanobody and analyzed by Western blot with anti-Mettl16 antibody. Only non-specific bands are detected in the Input lanes. In the GFP-IP lanes, the arrow indicates the Mettl16-GFP fusion protein. **(D)** Northern blot for snRNAs. In the RNA-IP sample, U6 is detected only from *Mettl16-GFP* flies, not from untagged *w¹¹¹⁸* controls. **(E)** Viability and fertility analysis of the indicated genotypes.

Given that METTL16 catalyzes m⁶A modification of U6 snRNA across multiple species, we next focused on U6 in *Drosophila*. Although our MeRIP-seq library was constructed from poly(A)-selected mRNA, we still detected clear m⁶A peaks on U6 snRNAs, and these peaks were substantially reduced in *Mettl16* mutants (Fig 6A, compare Mettl16¹*^–^*⁹-IP with w¹¹¹⁸-IP). To validate this result, we performed an independent m^6^A-immunoprecipitation (IP) Northern blot. Total U6 levels were unchanged in the mutants (Fig 6B, input), but the amount of U6 precipitated by an anti-m⁶A antibody was lower in *Mettl16*^*1*^*^–^*^*9*^ and *Mettl16*^*Δ*^*³³* than in *w*^*1118*^ controls (Fig 6B, anti-m^6^A).

We further asked whether Mettl16 physically associates with U6 snRNA *in vivo* using our Mettl16-GFP knock-in line. GFP immunoprecipitation followed by Northern blotting for U6 was performed. We first confirmed the specific recovery of the Mettl16-GFP fusion protein: while many non‑specific bands were present in the input, the anti-Mettl16 antibody detected a single band of the expected size after GFP‑IP (Fig 6C). Importantly, U6 snRNA was readily detected in the GFP-IP samples from *Mettl16-GFP* flies, but not from untagged *w¹¹¹⁸* controls (Fig 6D). These findings confirm that the Mettl16 protein interacts with U6 snRNA in vivo.

Next, we tested the genetic interaction between *Mettl16* and U6 using a deficiency (Df(3R)Exel7357) covering all three U6 snRNAs. While heterozygous *Mettl16¹^–^⁹*/Cyo; *Df(3R)Exel7357*/ + flies appear normal, removing one copy of U6 snRNAs turns *Mettl16¹^–^⁹/Mettl16¹^–^⁹* into lethality (Fig 6E). Collectively, these results indicate that Mettl16 interacts with U6 snRNAs physically and genetically.

### Mettl16 depletion broadly affects RNA splicing events

**Fig 7:**
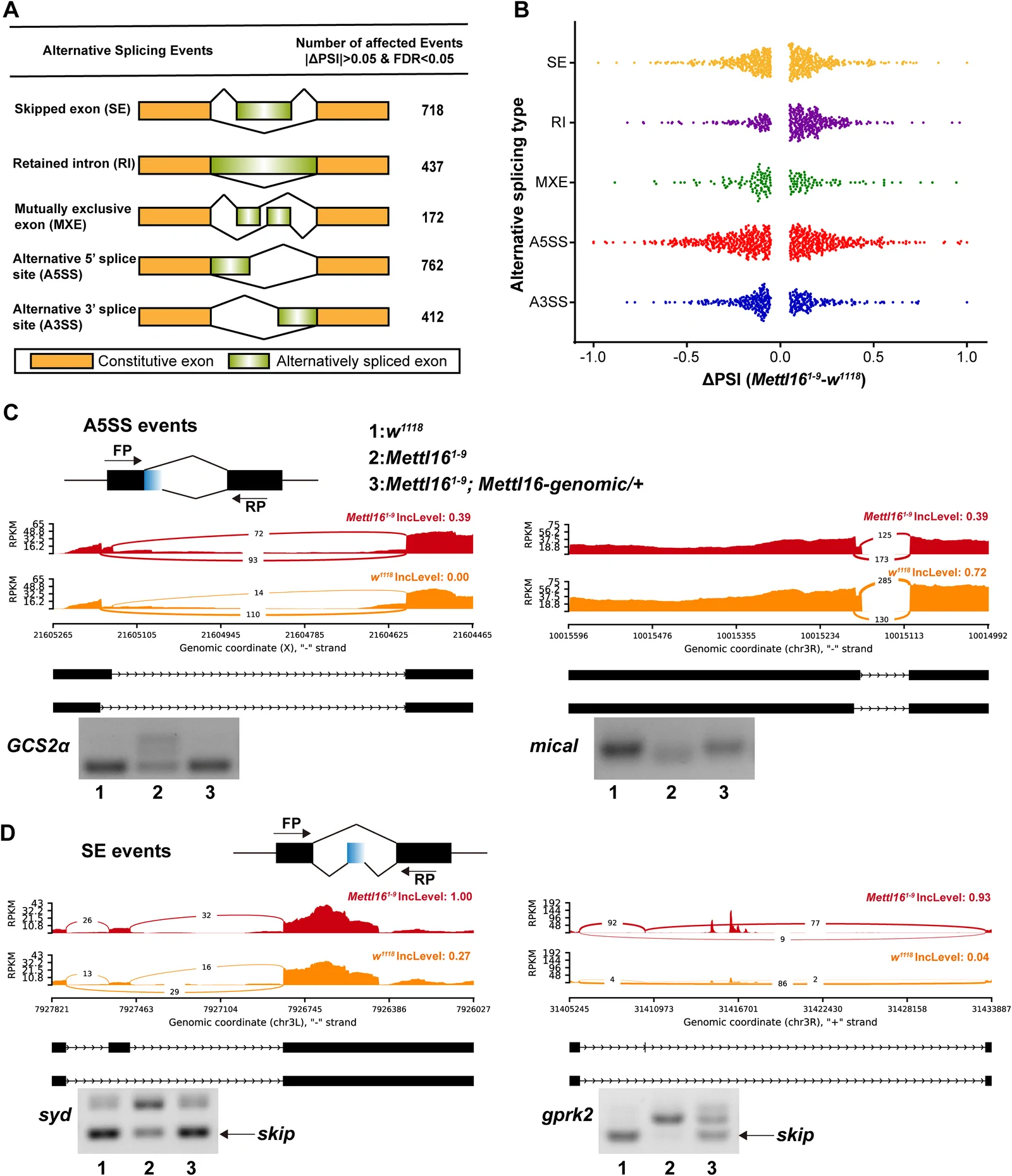
Global splicing alterations in *Mettl16* mutants. **(A)** Types and numbers of differential alternative splicing (AS) events in *Mettl16¹*^–^*⁹* versus *w¹¹¹⁸* (FDR < 0.05, |ΔPSI| > 0.05). ΔPSI = PSI_ *Mettl16¹*^–^*⁹* – PSI_ *w¹¹¹⁸*. **(B)** Distribution of ΔPSI for all significant AS events (FDR < 0.05 and |ΔPSI| > 0.05). Each point represents one event. **(C, D)** Experimental validation of representative A5SS (C) and SE (D) events. Top: Schematics of primer design (FP/RP) spanning the alternative region (blue). Middle: Sashimi plots from RNA‑seq (red, *Mettl16¹*^–^*⁹*; orange, *w¹¹¹⁸*) illustrate altered splice‑junction usage. Bottom: RT‑PCR confirmation using the same templates (1–3) indicated in **(C)**; gene names are shown on the left.

Having established that Mettl16 mediates the m^6^A modification on U6 snRNA, we next asked how its loss affects global splicing. Analysis of our RNA-Seq data with rMATS revealed widespread differential alternative splicing in *Mettl16*^*1*^*^–^*^*9*^ flies compared to *w*^*1118*^ controls, encompassing skipped exons (SE), retained introns (RI), mutually exclusive exons (MXE), alternative 5’ splice sites (A5SS), and alternative 3’ splice sites (A3SS) (Fig 7A and S6 Table). Consistent with prior models in which reduced U6 m^6^A impairs its pairing with the 5′ splice site, A5SS events were the most frequent change, followed by SE events. We selected several differential A5SS and SE events and validated them by RT-PCR (Fig 7C and 7D, lanes 1–2). Importantly, a genomic rescue transgene restored normal splicing patterns for most genes (Fig 7C and 7D, lane3), confirming that these defects are specifically caused by the *Mettl16* mutation. The splicing of one gene, *gprk2,* exhibits only a partial reversion to the normal pattern (Fig 7D). *gprk2* has thirteen exons and a complex splicing pattern and is expressed in diverse tissues; it is likely that *gprk2* requires a full dose of Mettl16 to achieve proper splicing. Furthermore, ΔPSI analysis showed that most RI events had positive values, indicating increased intron retention in the mutant (Fig 7B).

Recently, the *Drosophila* protein Amus, a homolog of mammalian MePCE, was shown to cap U6 snRNA and regulate its stability [49]. Interestingly, Amus associates with Mettl16 in previous high-throughput *Drosophila* Protein interaction Map (DPiM) [50]. A hypomorphic allele of *Amus* causes sterility in both male and female flies. In S2 cells, both GFP‑Mettl16 and mRFP‑Amus localize to the nucleus (S7B Fig). To identify potential interaction partners of *Drosophila* Mettl16, we transfected S2 cells with constructs expressing GFP‑Mettl16 or GFP‑myc (control) and performed affinity purification followed by mass spectrometry. Our data identified Amus as a top interactor of Mettl16 (S7A Fig, S7 Table). We then analyzed a series of intron retention events known to be regulated by Amus through RT-PCR [49]. Notably, RT‑PCR analysis showed that a set of intron‑retention events previously linked to *Amus* mutation were similarly altered in *Mettl16*^*1*^*^–^*^*9*^ mutants, and these defects were also restored by the *Mettl16*-genomic transgene (S7C Fig). Together, these results suggest that Mettl16 regulates a broad spectrum of alternative splicing events likely via its modification of U6 snRNA. These widespread splicing defects may underline the pleiotropic phenotypes observed in *Mettl16* mutants.

## Discussion

METTL16 is an evolutionarily conserved methyltransferase found in organisms ranging from bacteria and plants to mammals. Here, we present a functional analysis of its role in *Drosophila* development. Unlike the essential requirement for METTL16 in mouse embryonic development and cancer cell survival [11,51], *Drosophila Mettl16* mutants are viable. However, they exhibit multiple developmental and reproductive defects, including developmental delay, abnormal wing posture, reduced locomotor activity, flightlessness, and sterility in both sexes. Our genetic and cell-biological analyses reveal that spermatogenesis in the mutant proceeds normally through mitosis and meiosis, but is severely impaired at the elongation and individualization stages. RNAi knockdown in either germline or reproductive somatic tissues generates a milder phenotype than the mutant, suggesting that *Mettl16* is required in multiple tissues.

Interestingly, while mouse *METTL3* knockouts are embryonic lethal [52], *Drosophila Mettl3* mutants are also viable, suggesting that the essential functions of these enzymes may have diverged during evolution. *Drosophila Mettl3* mutants also exhibit abnormal wing posture, reduced locomotor activity, and flightlessness [34,35]. The “held-out wing” phenotype is shared by several *Drosophila* mutants affecting distinct aspects of wing/thorax development. For instance, alleles of *dpp* that disrupt the 3’ cis-regulatory *dpp*^*heldout*^ enhancer produce held-out wings [53,54], while *how* (*held out wings*) encodes a KH-domain RNA-binding protein required in muscle development [55,56]. Recent work shows that Mettl3 is required in both muscle and neurons, possibly through regulation of many different genes [57]. It will be interesting to dissect and compare the mechanisms of Mettl3- and Mettl16-dependent behavior defects in the future.

We investigated several previously proposed molecular functions of Mettl16. First, Mettl16-GFP is primarily localized to the nucleus rather than the cytoplasm. In line with this, depletion of *Mettl16* did not affect global translation in wing disc cells. Second, under normal dietary conditions, we found no evidence for Mettl16-dependent m^6^A regulation of *Sams* mRNA, consistent with observations in *Arabidopsis* [28]. It is worth noting that, in *C. elegans*, the METTL16 homolog METT-10 deposits m^6^A at the 3′ splice site under high-nutrient conditions, thereby inhibiting splicing. Third, quantitative LC-MS and MeRIP-seq analyses confirmed that Mettl16 is required for m^6^A modification of a small subset of transcripts. Whereas 2460 m^6^A peaks were significantly decreased identified in *Mettl3* mutants, only 186 were reduced in *Mettl16* mutants. Among these, 27 genes showed both decreased m^6^A peak intensity and altered expression levels, warranting further study.

We demonstrated that the total U6 level remains unchanged in *Mettl16* mutants, which is consistent with their viable yet mild phenotype. Mettl16 is necessary for m^6^A modification of U6 snRNA and interacts with U6 both physically and genetically. Previous work has shown that the m^6^A mark at U6 enhances splicing efficiency specifically for a subset of pre-mRNAs, which bear an A at the + 4 position of the 5′ splice site and contain exon sequences weakly recognized by U5 snRNA [30]. Certain tissues, such as the reproductive and nervous systems, might be enriched for m^6^A-sensitive targets, making them more vulnerable to Mettl16 loss. The synthetic lethality with heterozygous U6 deficiency supports the partial abrogation of U6 snRNA function in *Mettl16* mutants. When U6 dosage is reduced by 50%, the combined stress of a reduced U6 pool plus loss of m^6^A-mediated splicing fidelity exceeds the functional threshold of the spliceosome, resulting in lethality. Collectively, these findings suggest that the pleiotropic phenotypes of *Mettl16* mutants likely arise not from mis-regulation of one or a few genes, but from the cumulative impact of m^6^A loss on both U6 snRNA-dependent splicing and the m^6^A modifications of other target mRNAs.

## Materials and Methods

### Fly strains

Flies were maintained on standard cornmeal medium. The following stocks were used: *w*^*1118*^ (Bloomington 5905, wild-type control), *Df(2R)Exel7135* (*Mettl16* deficiency, Bloomington 7879), *Df(3R)Exel7357* (U6 snRNA deficiency, Bloomington 7948), *nos-Cas9* (Bloomington 54591), *dj-GFP* (Bloomington 58406), *hh-GAL4* [58], *en-GAL4, UAS-GFP* [58], *nanos-GAL4:VP16* (from Zhouhua Li), *tj-GAL4* (from Zhaohui Wang), *UAS-Rheb* (Bloomington 9689), *PPP1R15* RNAi (VDRC 107545). All experiments were performed at 25°C, except that the *nanos-GAL4:VP16* and *tj-GAL4* experiments were conducted at 29°C to enhance RNAi efficiency.

Two UAS-*Mettl16* RNAi lines were generated using the pNP vector in Tsinghua Fly Center. The line TH15374.S (target sequence: TTGATACTGCAACTGTTGCTG) was inserted at the attP40 site, and TH14423.S (target sequence: TAACACATTACGTGGATGCAT) at attP2.

For CRISPR mutagenesis, two sgRNA transgenic lines were constructed. A single sgRNA with the sequence TGCGTGCGTAACACATTACG was cloned into pCFD3.1 vector (Addgene 123366). Double sgRNAs with the sequences TGCGTGCGTAACACATTACG and ACGTTTCCACACCTTTGAGC were cloned into pCFD5_w vector (Addgene 112645). Both constructs were integrated into the attP40 site by PhiC31-mediated transformation at UniHuaii. These sgRNA lines were crossed to *nanos-Cas9* to generate mutations alleles; *Mettl16*^*1*^*^–^*^*9*^ and *Mettl16*^*Δ33*^ were selected for subsequent analysis.

### Clonal analysis

Mitotic clones were generated by heat-shock-induced FLP-FRT recombination. *Mettl16* ^*1*^*^–^*^*9*^ *FRTG13* flies were crossed to *hs-flp; ubi-GFP, FRTG13* flies, and progeny were heat-shocked at 37 °C for 1 h twice at first- and second-instar larval stage. Third-instar larvae were dissected and stained.

### Transgenic constructs

The *Mettl16‑GFP* strain was generated by CRISPR‑Cas9‑mediated homologous recombination. Two sgRNAs (CACTCGGAGCCACTACAGCA and AATTAAGTTACCATTAGCTC) targeting sequences flanking the *Mettl16* stop codon were designed. A donor plasmid was constructed by cloning a 5′ homology arm (~1.5 kb upstream of the stop codon), the GFP coding sequence, and a 3′ homology arm (~1 kb downstream) into pBluescript SK(–) (primers in S8 Table). sgRNAs and Cas9 mRNA were synthesized by in vitro transcription. A mixture of sgRNAs, Cas9 mRNA, and donor plasmid was injected into *w¹¹¹⁸* embryos at Fungene. Knock-in flies were identified by PCR and confirmed by DNA sequencing (complete sequence shown in S1 Text).

For the *UAS-Mettl16* fly, the *Mettl16* coding sequence was inserted into the pWALIUM10-roe vector, and the construct was integrated into the attP40 site at UniHuaii.

For the *Mettl16* genomic rescue fly, a 3737 bp DNA fragment (chr2R: 15146987–15150723; Release 6.54) containing about 1kb upstream and 1.6kb downstream of *Mettl16* coding region was cloned into the pUAST-attB vector, and the construct was integrated into the attP2 site at UniHuaii.

### RT-PCR

Total RNA was extracted from 10–15 adult male flies using TRIzol reagent (Invitrogen, 15596018). cDNA was synthesized using Hifair II 1st Strand cDNA Synthesis SuperMix with gDNA digester plus (Yeasen, 11123). Regular PCR was performed with 2 × Hieff PCR Master Mix (Yeasen, 10102), and quantitative PCR (qPCR) with Hieff qPCR SYBR Green Master Mix (Yeasen, 11201). All primers are listed in S8 Table; primers used in S7 Fig were as described in reference [49].

### Western Blot

Fly samples were homogenized and lysed in ice‑cold NP‑40 lysis buffer (Beyotime, P0013F) supplemented with PMSF, and incubated on ice for 1 h with brief vortexing every 5 min. The lysate was centrifuged at 12,000 × g for 15 min at 4 °C, and the supernatant was collected. After mixing with 5 × SDS loading buffer and boiling at 95 °C for 2 min, proteins were separated by SDS‑PAGE, transferred to a PVDF membrane (Millipore, IPVH00010), and immunoblotted with anti‑α‑tubulin (Sigma, T9026, 1:5000) or with a custom rabbit anti‑Mettl16 (E20681, 1:1000). This antibody was raised against recombinant full‑length Mettl16 (aa 1–305) and affinity‑purified at ABclonal.

### Developmental timing and climbing assay

Flies were mated and transferred to fresh vials every 24 hours. Pupariation was scored every 12 hours beginning at 96 hours post-mating, and adult eclosion was scored every 12 hours beginning at 216 hours. Pupariation and eclosion rates were calculated as the cumulative number of individuals at each time point divided by the final total.

Locomotor ability was assessed using a negative geotaxis assay. For each genotype, flies were placed in vials (15 flies per vial, 6 vials). A line was marked at 5 cm height. Flies were tapped to the bottom, and the number that crossed the line within 5 seconds was recorded.

### Immunostaining and microscopy

Tissues (third‑instar wing discs, adult testes/ovaries) were dissected in ice-cold PBS, fixed in 4% paraformaldehyde (Beyotime, P0099) for 15 min (wing discs) or 30 min (gonads) at RT respectively, and washed in 0.1% PBST (PBS + 0.1% Triton X-100). After blocking in 5% normal donkey serum (Jackson Immuno, 017-000-121) in PBST, samples were incubated with primary antibody overnight at 4 °C, washed, and incubated with secondary antibody for 2.5 h at room temperature (RT) in the dark. After final washes, tissues were mounted in antifade medium (Beyotime, P0126) and imaged on an Olympus FV‑3000 confocal microscope. Antibodies used: rabbit anti-GFP Alexa Fluor 488 (1:800) (Invitrogen, A21311), rabbit anti-cleaved Caspase-3 (1:400) (Cell Signaling Technology, 9661), rabbit anti-Vasa (1:500) (Santa Cruz Biotechnology, d-260), rabbit anti-Histone H3 (1:1000) (Cell Signaling Technology, 9715), mouse anti-Orb (1:50) (DSHB, 6H4 and 4H8, 1:1), mouse anti-Eya (1:200) (DSHB, 10H6), Alexa Fluor 488 phalloidin (1:1000) (Invitrogen, A12379), and DAPI (1:1000) (Beyotime, C1002).

Testes were dissected from adult male flies at 3 days post-eclosion and prepared for transmission electron microscopy according to standard protocols. Ultrathin sections were examined and imaged using a JEOL1400Flash transmission electron microscope.

### OPP assay for protein synthesis

Protein synthesis was measured with the Click-iT Plus OPP Protein Synthesis Assay Kit (Thermo Fisher, C10457) [45]. Third-instar larvae were dissected in Schneider’s *Drosophila* medium (Gibco, 21720024) at RT and then incubated in 1μM OPP reagent in Schneider’s medium for 15min with shaking. Samples were briefly rinsed with PBS, fixed in 4% paraformaldehyde (Beyotime, P0099) for 15 min, and permeabilized in PBST for 15 min. The Click-iT reaction was performed for 30 min in the dark. Samples were rinsed with the Reaction Rinse Buffer, mounted in antifade medium, and imaged on a confocal microscope.

### Analyzing m^6^A levels by LC-MS

Total RNAs were extracted from adult flies with TRIzol (Invitrogen, 15596018) and subjected to two rounds of poly(A) selection using the GenElute mRNA Miniprep kit (Sigma, MRN10). RNA was hydrolyzed to ribonucleosides with nuclease P1 (Sigma, N8630) and snake venom phosphodiesterase (Sigma, P3134) at 37°C for 2h, followed by digestion with fast alkaline phosphatase (Thermo Fisher, EF0654) for an additional 2h.

Quantitative LC-MS was performed on a SCIEX Exion system coupled to a SCIEX QTRAP 6500 + mass spectrometer operated in positive-ion mode with dynamic multiple reaction monitoring. Ribonucleosides were separated on a Zorbax Eclipse Plus C18 column (Agilent Technologies) at 300 μl/min using 0.2 mM NH_4_HCO_3_ (A) and methanol (B) as mobile phases. Nucleosides were detected via the transition from the parent ion to the deglycosylated base ion: *m/z*268 → 136 for adenosine (A) and *m/z*282 → 150 for m⁶A. Absolute quantities were determined using external calibration curves prepared with A (Sigma, A9251) and m^6^A (Selleck, S3190).

### MeRIP-seq

Total RNA was isolated and purified using TRIzol reagent from ~50 adult males of *Mettl16*^*1*^*^–^*^*9*^ and *w*^*1118*^. The concentration and purity of each RNA sample were quantified using a ND-1000 spectrophotometer (Nano-Drop), while RNA integrity was assessed via an Agilent 2100 Bioanalyzer (Agilent). Poly(A) RNA was purified from 50 μg of total RNA using Dynabeads Oligo(dT) (Thermo Fisher, 25–61005) through two rounds of purification. The purified RNA was fragmented into small segments using the Magnesium RNA Fragmentation Module (NEB, E6150) at 86 °C for 7 min. The fragmented RNA was then incubated with Dynabeads Antibody Coupling Kit (Thermo Fisher) and an m^6^A-specific antibody (Synaptic Systems, 202003) in IP buffer (50 mM Tris-HCl, 750 mM NaCl, and 0.5% Igepal CA-630) at 4 °C for 2 h.

The immunoprecipitated (IP) RNA was reverse-transcribed into cDNA using Super Script II Reverse Transcriptase (Invitrogen, 1896649). Subsequently, U-labeled second-stranded DNAs were synthesized from the cDNA using Escherichia coli DNA polymerase I (NEB, M0209), RNase H (NEB, M0297), and dUTP Solution (Thermo Fisher Scientific, R0133). A single A-base was added to the blunt ends of the double-stranded DNAs to facilitate ligation with indexed adapters. Dual-index adapters were then ligated to the A-tailed fragments, followed by size selection using AMPure XP beads. After treating the U-labeled second-stranded DNAs with heat-labile uracil-DNA glycosylase (UDG; NEB, M0280) to eliminate the U-labeled strand, the ligated products were amplified by polymerase chain reaction (PCR) under the following conditions: initial denaturation at 95 °C for 3 min; 8 cycles of denaturation at 98 °C for 15 s, annealing at 60 °C for 15 s, and extension at 72 °C for 30 s; and a final extension at 72 °C for 5 min. Finally, paired-end sequencing (PE150) was performed on an Illumina NovaSeq 6000 platform (LC-Bio Technology) in accordance with the vendor’s recommended protocol.

### Bioinformatic analysis

fastp software was used to remove the reads that contained adaptor contamination, low quality bases and undetermined bases with default parameter. And sequence quality of IP and Input samples were verified using FastQC and RseQC. Then we used HISAT2 to map reads to the reference genome *Drosophila melanogaster* (Version: v107). Peak calling and diff peak analysis were performed by R package exomePeak, and peaks were annotated by intersection with gene architecture using ANNOVAR. HOMER was used for de novo and known motif finding followed by localization of the motif with respect to peak summit. And StringTie was used to perform expression level for all transcripts and genes from input libraries by calculating FPKM (total exon fragments/mapped reads (millions) × exon length (kB)). The differentially expressed transcripts and genes were selected with log2 (fold change) ≥ 1 or log2 (fold change) ≤ -1 and p value < 0.05 by edgeR. rMATS was used for differential splicing analysis.

### RNA-IP and Northern blot

RNA immunoprecipitation (RNA-IP) was performed as described previously [59]. Briefly, 20 flies (1-2d old) of the *Mettl16-GFP* or *w¹¹¹⁸* strain were homogenized and lysed in NP40 buffer supplemented with RNase OUT (Invitrogen, 10777019; 400 U/mL). An aliquot of lysate was saved as input; and the remainder was incubated with GFP‑Trap agarose (Chromo Tek, gta-20) overnight at 4 °C. After washing twice with ice‑cold PBS, the bead‑bound material was split. One portion was eluted in buffer containing 50 mM Tris-HCl (pH 7.0), 5 mM EDTA, 10 mM DTT, 2% SDS, and analyzed by Western blot with anti-Mettl16 (E20681). The other portion was treated with proteinase K at 55 °C, and RNA was extracted with TRIzol for Northern blot.

For Northern blot, total RNA was denatured in RNA loading buffer (Thermo Fisher, R0641) at 70 °C for 5 min and separated on an 8% urea-polyacrylamide gel in 1 × TBE (Beyotime, R0223, then transferred to a nylon membrane (Cytiva, RPN303B) in 0.5 × TBE at 20 V for 30 min. After UV cross-linking, the membrane was pre-hybridized in ULTRAhyb-Oligo buffer (Invitrogen, AM8663) at 42 °C for 1h, then hybridized with 0.1 μg/mL biotinylated probes overnight at 42 °C. The membrane was washed twice (30min each) with 2 × SSC (Beyotime, ST462) containing 0.5% SDS (Beyotime, ST629) at 42 °C and processed using the Chemiluminescent Nucleic Acid Detection Module kit (Thermo Fisher, 89880) according to the manufacturer’s instructions.

### m^6^A-IP

m^6^A immunoprecipitation (m^6^A-IP) was performed as described [60]. Briefly, 15μg total RNA extracted from *w*^*1118*^, *Mettl16*^*1*^^*–*^^*9*^, or *Mettl16*^*Δ33*^ flies was resuspended in IP buffer (50 mM Tris-HCl (pH 7.4), 750 mM NaCl and 0.5% Igepal CA-630), denatured at 70 °C for 5 min, and snap‑cooled on ice. The RNA was incubated overnight at 4 °C with 5μg of anti-m^6^A antibody (Synaptic Systems, 202003) in IP buffer supplemented with 0.4 U/μl RNasin. A control IP was run in parallel using rabbit IgG (Beyotime). 30μL Dynabeads Protein A (Thermo, 10001D) was added, and the mixture was incubated for 2h at 4 °C with rotation. Beads were washed three times with ice‑cold IP buffer, and bound RNA was eluted with 6.7mM N⁶‑methyladenosine 5′‑monophosphate sodium salt (Sigma‑Aldrich, M2780) in IP buffer. Eluted RNA was recovered by TRIzol extraction and analyzed by Northern blot as above.

### Cell culture

To generate GFP- and mRFP-tagged plasmids, cDNAs of *Mettl16* and *Amus* were first cloned into the pENTR vector (Invitrogen) and then recombined into the *Drosophila* Gateway destination vectors pAGW and pARW using Gateway LR Clonase II Enzyme Mix (Invitrogen, 11791–020). *Drosophila* S2 cells were maintained in Schneider’s medium (Gibco, 21720024) supplemented with 10% fetal bovine serum (Gibco, 10099141C) at 25 °C. Cells were seeded in 100mm dishes and transfected with 4μg of plasmids using Effectene Transfection Reagent (QIAGEN, 301425). After 48h, cells were transferred to glass-bottom confocal dishes (Biosharp, BS-15-GJM) for live imaging.

### IP-MS

S2 cells expressing GFP‑Mettl16 or GFP‑myc [37] were harvested 48 h post‑transfection, washed with PBS, and lysed in NP-40 buffer. Target proteins were purified using GFP‑Trap agarose (ChromoTek, gta‑20), denatured, and subjected to three rounds of buffer exchange with 8 M urea/100 mM Tris‑HCl (pH 8.0). Samples were reduced with 10 mM DTT at 37 °C for 30 min, alkylated with 30 mM iodoacetamide at 25 °C for 45 min in the dark, and buffer‑exchanged three times with 30 mM Tris‑HCl (pH 8.0). Proteins were digested with trypsin (1:50 enzyme‑to‑protein ratio) at 37 °C for 12 h. Digested peptides were filtered, dried, and reconstituted in 15% acetonitrile.

MS analysis was performed on a nanoflow EASY-nLC 1200 system (Thermo Fisher) coupled to an Orbitrap Exploris 480 mass spectrometer (Thermo Fisher). Peptides were separated on a C18 column (75 µm × 25 cm, 1.9 µm) using a 60‑min gradient of 0.1% formic acid in water (A) and 0.1% formic acid in 80% acetonitrile (B) at 200 nL/min. High-field asymmetric-waveform ion mobility spectrometry (FAIMS) was applied with compensation voltages of −40 V and −60 V. MS1 spectra were acquired at a resolution of 60,000. MS2 spectra were collected at 15,000 resolution with HCD fragmentation (NCE 30%), using a 1.6‑Da isolation window, 45‑s dynamic exclusion, and 1‑s cycle time. Data were searched against the *Drosophila melanogaster* protein database.

## Supporting information

S1 Text Sequence of *Mettl16-GFP* knock-in flies. (DOCX)

S1 Fig *Mettl16* mRNA and protein expression levels in mutants. (A) qPCR analysis of *Mettl16* mRNA levels in total RNA from adult male flies. (***P < 0.001). (B) Western blot of Mettl16 protein in lysates from adult flies and third-instar larvae. The arrow indicates the band corresponding to Mettl16.
(TIF)

S2 Fig Mitotic and meiotic stages are normal in *Mettl16* mutant testes. (A-B) Bright-field images of testes. Elongated flagella can be observed in both control group and *Mettl16*^*1*^*^–^*^*9*^ mutant testes (yellow arrows). Vasa (A’-B’) and Histone H3 (C-D) antibody staining. Both control and the *Mettl16*^*1*^*^–^*^*9*^ mutants progress normally through the mitotic 8- and 16-cell stages (Vasa, red and yellow outlines) and the meiotic 32- (Vasa, white outline) and 64-cell stages (round spermatid) (H3, white outline). Scale bars: 50 μm.
(TIF)

S3 Fig Mettl16 acts in both germline and somatic cells of the reproductive system. (A-A”’) Testes of *Mettl16-GFP* flies stained for Eya (red) and DAPI (blue). Mettl16-GFP is detected in Eya-positive cyst cells. (B-E”) Ovaries stained for Orb (red) and DAPI (blue). *Mettl16* knockdown by *tj-GAL4* or *nanos-GAL4:VP16* leads to defects including mislocalized Orb and abnormal germ cell numbers (arrows in B,” C,” D” and E”). (F-I”) Testes stained for cleaved Caspase-3 (red) and phalloidin (green). *Mettl16* knockdown by *tj-GAL4* or *nanos-GAL4:VP16* results in the absence of waste bags (arrows in F, G, H and I) and dispersed actin cones (arrows in F’, G’, H’ and I’). Scale bars: 50 μm.
(TIF)

S4 Fig Global translation in wing discs is unaffected by Mettl16 depletion or overexpression. (A-F”) OPP incorporation assay (red) in wing discs expressing the indicated transgenes by *en-GAL4, UAS-GFP* (posterior region marked by GFP, green). (A-A”) control (*en-GAL4, UAS-GFP*). (B-B”) Overexpression of Rheb robustly increases OPP incorporation. (C-C”) *PPP1R15* RNAi knockdown reduces OPP staining. (D-E”) Knockdown of Mettl16 using two independent RNAi lines does not affect OPP incorporation. (F-F”) Overexpression of Mettl16 does not alter OPP levels. (G-G”) Clonal analysis shows that OPP levels are unchanged in *Mettl16*^*1*^*^–^*^*9*^ mutant clones. Scale bars: 50 μm.
(TIF)

S5 Fig Differential m^6^A peaks and differential gene expression in *Mettl16* mutants. (A, B) GO (A) and KEGG (B) enrichment analysis of genes with differential m^6^A peaks in *Mettl16*^*1*^*^–^*^*9*^ mutants. (C) Volcano plot of differentially expressed genes in *Mettl16*^*1*^*^–^*^*9*^ mutants versus *w*^*1118*^ controls. A total of 610 genes were upregulated and 809 were downregulated (|log2FC| > 1, P < 0.05). (D) Gene Ontology (GO) enrichment analysis of the differentially expressed genes (Top 20 terms ranked by P-value).
(TIF)

S6 Fig Analysis of *Sams* m^6^A modification and expression in *Mettl16* mutants. (A) IGV snapshot of MeRIP-seq (red tracks, IP) and RNA-seq (blue tracks, Input) coverage across the *Sams* locus in *Mettl16*^*1*^*^–^*^*9*^ and *w*^*1118*^ flies. An m^6^A peak in the 3’UTR (right arrow) persists in mutants. The arrow on the left indicates the location of the conserved tacagAAAC motif at the intron 4–exon 5 junction, where no m^6^A peak was detected. (B) Schematic of the *Sams* transcript (red boxes denote exons, as in panel A). qPCR analysis of the expression levels for the exon and intron regions indicated by the colored dashed lines.
(TIF)

S7 Fig Mettl16 colocalizes and interacts with Amus. Volcano plot of proteins identified by affinity purification-mass spectrometry with GFP-Mettl16 versus GFP control (red dots with log_2_FC > 3, P < 0.05). Proteins with a Sum PEP Score > 30 are shown. The Amus protein is a top candidate. (B) Live imaging of S2 cells co-transfected with GFP-Mettl16 and RFP-Amus, both of which showed nuclear localization. (C) RT-PCR validation of intron retention (RI) events in *Mettl16*^*1*^*^–^*^*9*^ mutants, assessing a set of splicing changes previously linked to *Amus* mutation. Schematic (left) shows primer design spanning the intron of interest. The shorter band represents the spliced product; the longer band corresponds to the retained intron.
(TIF)

S1 Table m^6^A peak calling in *w*^*1118*^ and *Mettl16*^*1*^*^–^*^*9*^ male adult flies. (XLSX)

S2 Table Differential m^6^A peaks in *Mettl16*^*1*^*^–^*^*9*^ male adult flies versus *w*^*1118*^. (XLSX)

S3 Table Gene expression in *w*^*1118*^ and *Mettl16*^*1*^*^–^*^*9*^ mal e adult flies analyzed by RNA-seq. (XLSX)

S4 Table Differential gene expression in *Mettl16*^*1*^*^–^*^*9*^ male adult flies versus *w*^*1118*^. (XLSX)

S5 Table Differential m^6^A peaks and differential gene expression in *Mettl16*^*1*^*^–^*^*9*^ male adult flies versus *w*^*1118*^. (XLSX)

S6 Table Differential spliced events categorized by A3SS, A5SS, MXE, RI, and SE in *Mettl16*^*1*^*^–^*^*9*^ (sample1) male adult flies versus *w*^*1118*^ (sample2). (XLSX)

S7 Table IP-MS results using GFP-Mettl16 and GFP-myc as baits. (XLSX)

S8 Table Sequences of primers used in this study. (XLSX)

S1 Raw Gel Original images for blots and gels. (PDF)
