# Supplementary material for: Role of the m6A methyltransferase Mettl16 in Drosophila development
Source: PLoS Genet. 2026 Sep 24;22(9):e1012317. doi: 10.1371/journal.pgen.1012317 (PMC13608003; doi:10.1371/journal.pgen.1012317)

Fig. 3

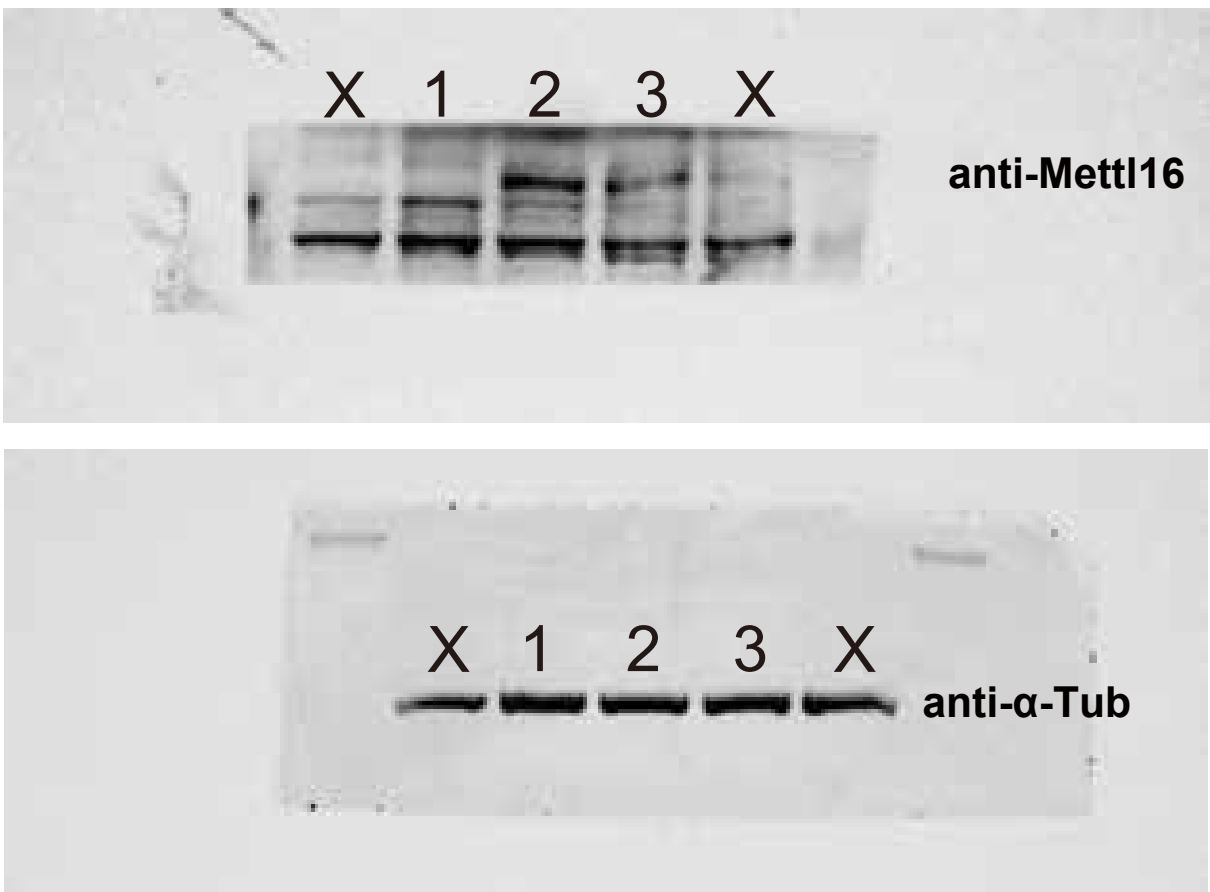

Fig. S1

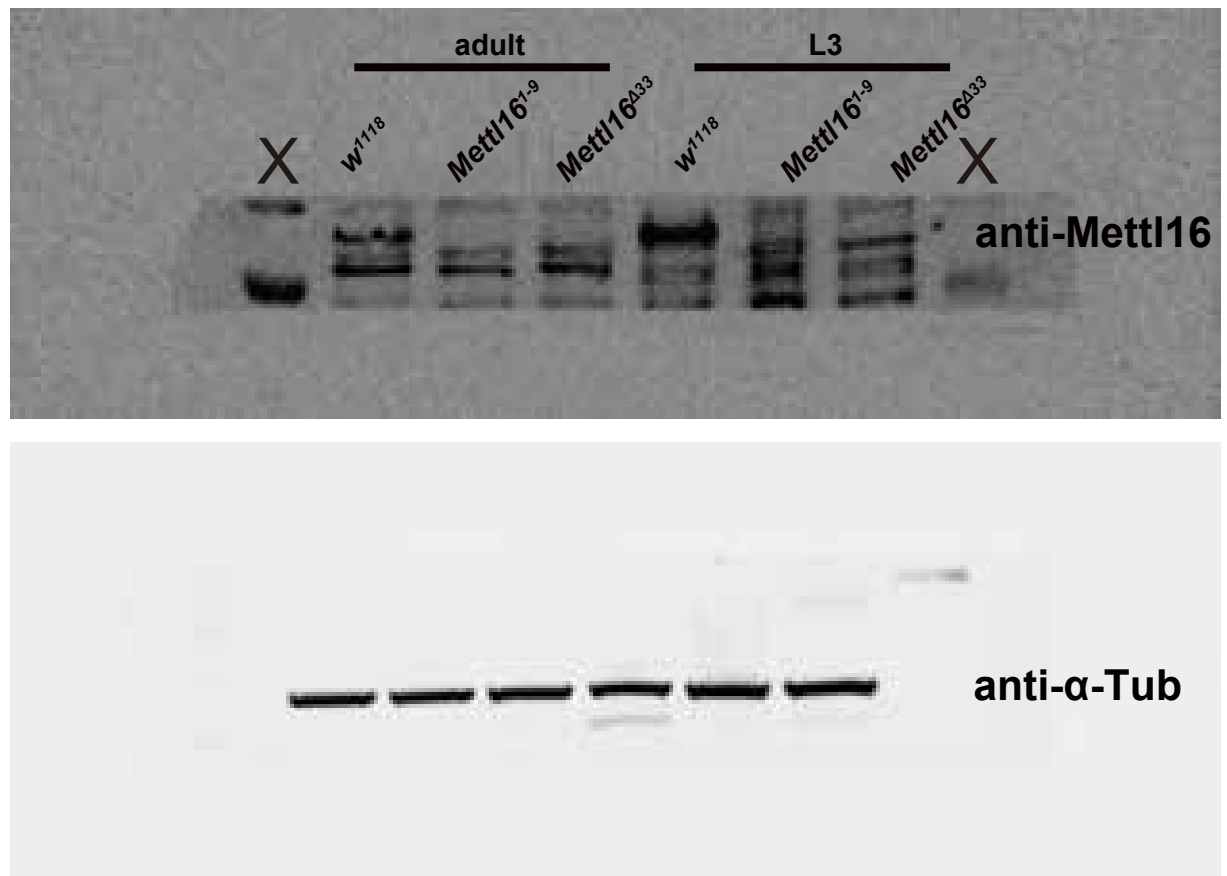

Fig. 6B

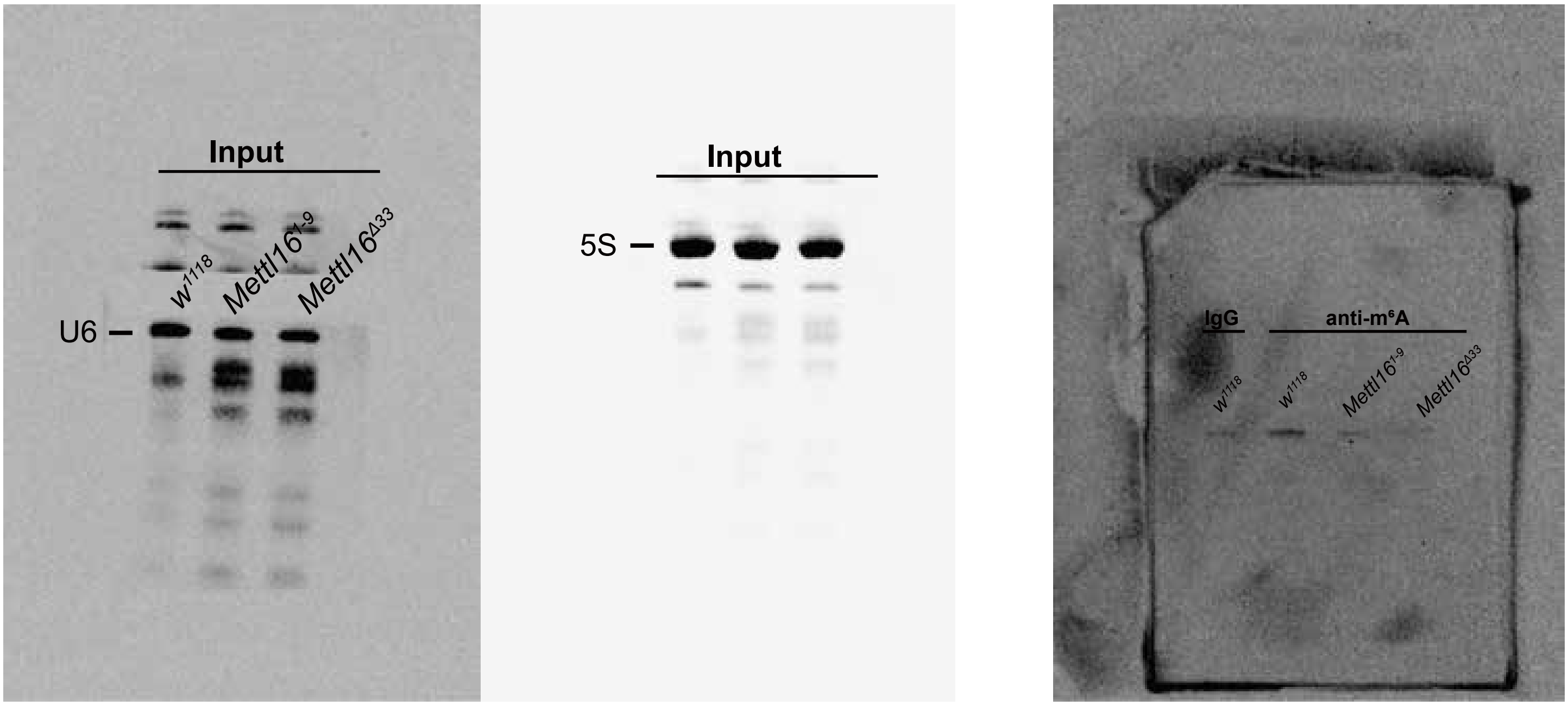

Fig. 6C

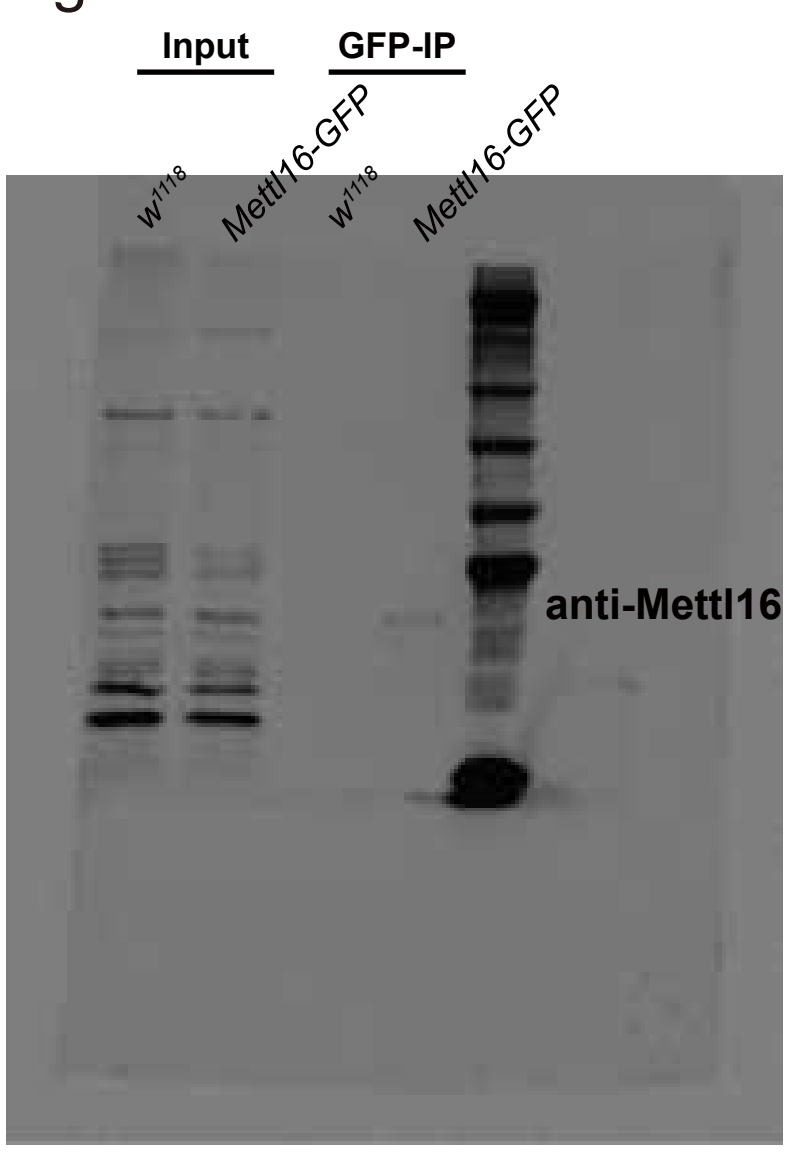

Fig. 6D

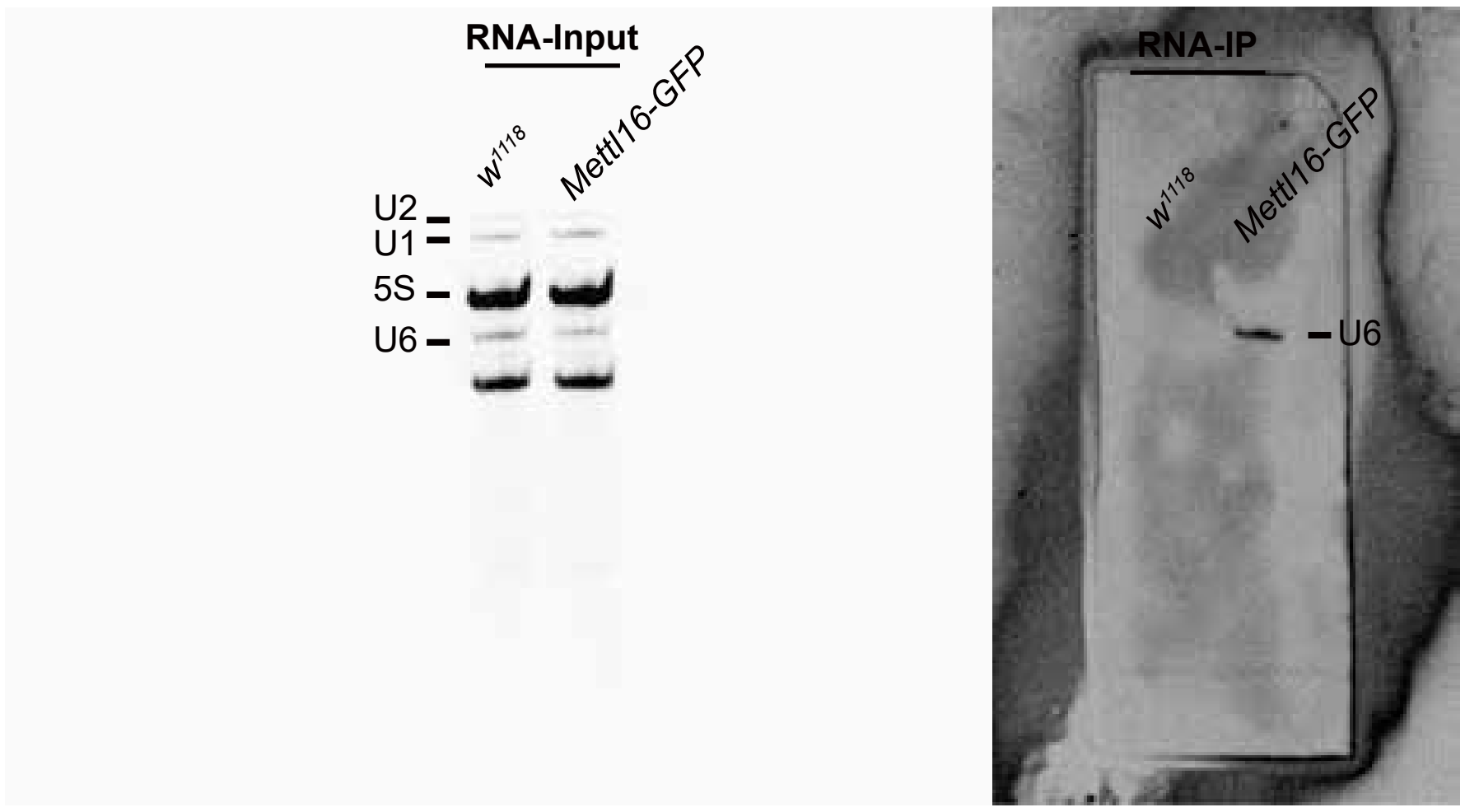

Fig. 7 and Fig. S7

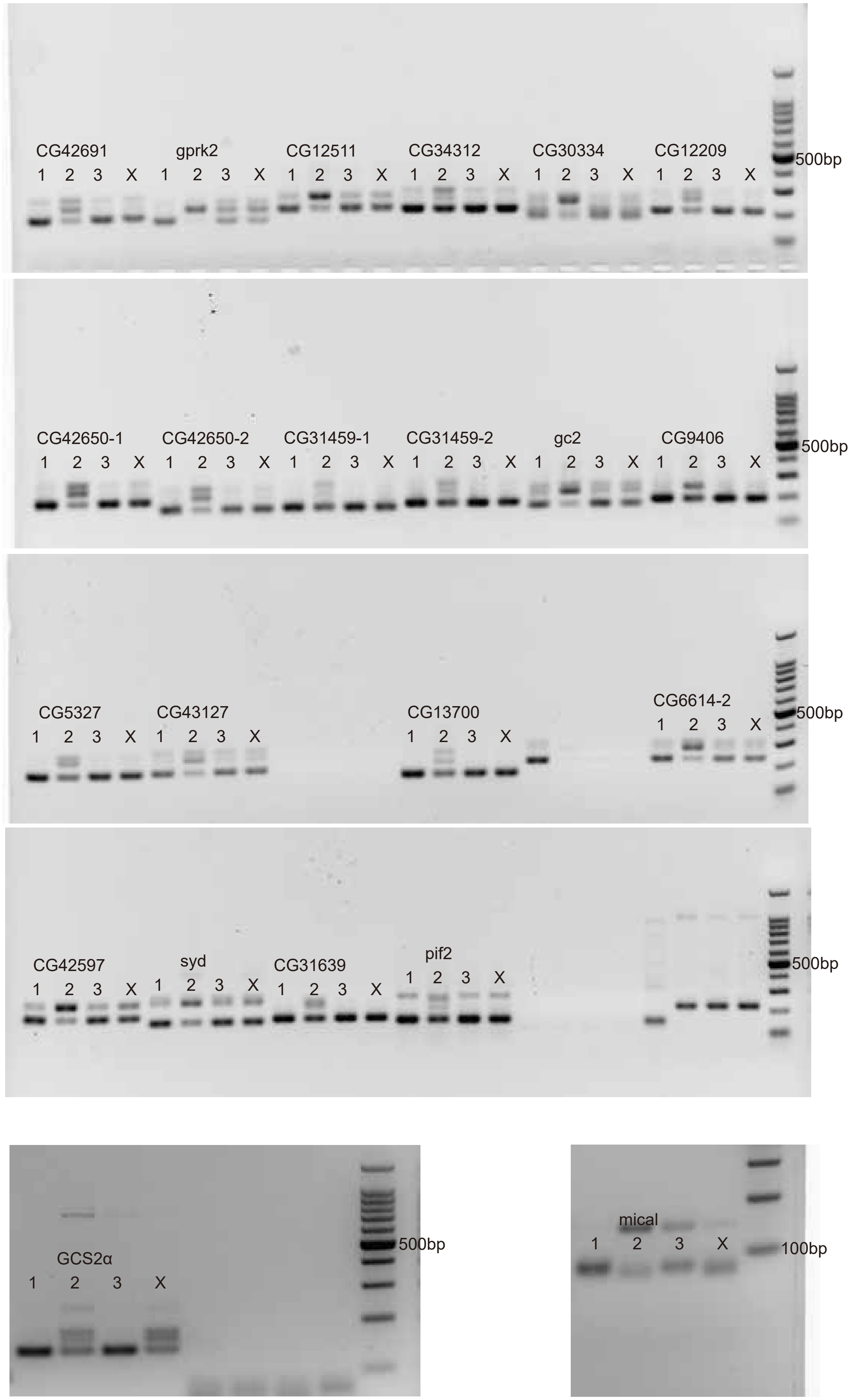

Supplement: Supplementary file 1 [file pgen.1012317.s017.pdf]
