# Supplementary material for: Role of the m6A methyltransferase Mettl16 in Drosophila development
Source: PLoS Genet. 2026 Sep 24;22(9):e1012317. doi: 10.1371/journal.pgen.1012317 (PMC13608003; doi:10.1371/journal.pgen.1012317)
Supplement: Supplementary file 2 [file pgen.1012317.s001.docx]

## **Genome sequence of KI line near by the target region**

AACCATATGCAGCTGGTGGCTAAAGGAGAAGCCATAGTTCTGAGCGCAACTTGGACATTTGCTGAGGCTAGAAGTCTTTCGTTTTTGAATTGGATGTTTCGATTCATCCTTAAAGTACGATTGCAGGTCCTCGGCAACTGGCACCAAGATATCCTCTACCTTGTTAAGTGATAGCTCCAGTTCCTTCTCCTCTTGTGACTTCTGATTCTCCAGTAGTCGTTTATATCTCTTGTTTTCGTGAAGTTCCCGCGAGCTTGTGGATTTTCTGATTATGTGTGAGGTTAACGAGTGCTGCAGATAGAATCGCCTGTAGCATATCTGGCAGTGAAAGTTGTCTGAATAGCGATGCGTCTTTTGATGATCCTCCACGTCCTGCAGCTTTTTCCACGGTAGATTCTCACATTTGTAGCAATCGCAGCGCAGTCCTGGTAGGAATAAAGCTGCAATTTTGGGACGCAGAGCTCCATGTGCTTCCTGGCCTGCCAGTTGGCTAACTGAACGTCTGTAGCCGCTGCTCACTGTATCCAGAATGGCAGCTAACTTTTCCAATTGTACTTTGCGGTTCTTCTCTTCGAAGGCATCGAATATTGCTCGAAAATCGTCCTGTAAATCAGCCTTAAAATACTCATTGGTCCAGAAATTATCGCGTAATTGTGCTGTGCGCAGTAATAGCAGCATATCCGAGCTTCGGAAACGCATAATTTTCTGAACGTCTTCGCATAACTCGCTGGAGAAATCCGTTCCAAATATGGTCTCCCCTTCCCCATTGAATGCCACGTACTTCTCCCTGAATCCTCGACAGCACTTGCACTTGCAGTGGCAGGAAGCAGGGCATCCATATGGCTTCTTGTCAATGCTAAGGACTGGCAAATCGGTTGTAAAGTTTTCGTAGGTAACTAGCAGTTTGCCAGGCCAAGACATTAAGTTTACTTTTTTGTTTACAAAAGTTGAATCAACAAGTGGCTGTGTGACCAGTCACTGATAGCACAGaagggccaaataaaactaccaattagaggagctggTGTCATCTCTTGCTCGCTGGAGTTGCCGGACTGCTGCAATAAACAGCTGTTCGAATTTCGGCGGCATTTTATTTCATTAACAATTGCGATTTTTTGAAAACGAAAT**ATG**GTAAAAACTAAGGGAAACCAAAAGATAGTCGGCATGCATCCACGTAATGTGTTACGCACGCAGCCCGACTATACCAAAATGGCTATAAAATACAAAGATTTTCGGCAGCAGTGCCAGTTGgtaagtaaaaactatttgttttgcttagacattgattttaataaaatattgcttaatttagGAGCTCAATGGCAAGGTTTCTGTGAACTTTCGCAATGAAAAGACCCTTCGTGAGTTGACCAAAATGCTGTTGAAGGAATATTACGATTTAGATGTGGATTTTGCACCCGGAAGTTTGGTGCCCACATTAGCACTACGTTTGAATTACATTTTGTGGTTGGAGGATCTAATGGAACCTCTAAATTTGCAGAACATACGTGGTATAGATATAGgtaagctatggttagtatgtcacaacgatttactaagtgaattcctcacagGCTGCGGCTCCTCCTGCATTTATTCACTCCTTGGGGCCAAGAAAAATGGTTGGCACATGTTGGCTTTGGAATCGAAACCGCAAAACATAGAATACGCCAAGGAGAATGTAAAGAGAAATCACATGGAAAGCCTTATAGAAGTCTATGCTCAACCAGACAACACGAACATCTTTAAGAGCTATTTTGAGCAGGATCAGCAACAGTTGCAGTATCAATTTTGCCTGTGCAATCCACCCTTCTTCGACTCGAACTTACCAAATCCCTTAGGCGGCAATACCAGGAATCCAGAGAGACGACCAGCACCCAATAATGCCAGAACTGGAAGCCAGGAGGAACTCACCTGCGTGGGTGGCGAGGTACAGTTTGTGCAACGCATTATAGACGAGAGTCTGGAGAACAAGGAGCGTGTGCGgtgagtcaatcttcattagctgctgagattgaattaacgttggttttctctgcgttgcagAATCTTCACCACGATGCTGGGCGTCAAGGCCAACGTTCCAAGAATACTGGATTACCTCAAGGAGCTGCAGGTGGCGAATGTCAGCACCACTGAATTCCATCAGGGACACACGACTCGGTGGGCGGTGGCCTGGAGCTTC**CACTCGGAGCCATTGCAACAGGG**GACAACAggTggAGGAGGCTCTGGTGGAGGCGGTAGCGGAGGCGGAGGGTCGgtgagcaagggcgaggagctgttcaccggggtggtgcccatcctggtcgagctggacggcgacgtaaacggccacaagttcagcgtgtccggcgagggcgagggcgatgccacctacggcaagctgaccctgaagttcatctgcaccaccggcaagctgcccgtgccctggcccaccctcgtgaccaccctgacctacggcgtgcagtgcttcagccgctaccccgaccacatgaagcagcacgacttcttcaagtccgccatgcccgaaggctacgtccaggagcgcaccatcttcttcaaggacgacggcaactacaagacccgcgccgaggtgaagttcgagggcgacaccctggtgaaccgcatcgagctgaagggcatcgacttcaaggaggacggcaacatcctggggcacaagctggagtacaactacaacagccacaacgtctatatcatggccgacaagcagaagaacggcatcaaggtgaacttcaagatccgccacaacatcgaggacggcagcgtgcagctcgccgaccactaccagcagaacacccccatcggcgacggccccgtgctgctgcccgacaaccactacctgagcacccagtccgccctgagcaaagaccccaacgagaagcgcgatcacatggtcctgctggagttcgtgaccgccgccgggatcactctcggcatggacgagctgtacaag**TAAAATTAAGTTACCATTAGCTCTCg**tagagtatctttaagatggtaggggagaagtagtttaacttattttggattttataagatttattcaataatttcatttttataggcatcagTTTTAATGTATAAGTTCTTGGAGAATTGCTGCAACTTTCGAATGTACTGTGACCTTCACCTGTGCTCATCCCAAAAAGCAACTCCAAGGCACCTAGAAAGCTTTCGCCTTAAATGGAAATTTGCATGTCAACGGTCACTTTGCCACCCAGCTCCACGAACGGAAATTATCGTTTCGCTTTTATCATTGACCACAGCTGACCACACACTTCGACGGAAAAAGGCAAACGGAGTCGAAATGGCAACCGAAATGGAGGCAAACACGCTCACTAAGCACAAGTTGCCGCCGCTGGTCAACACACCGACGTTTCCACACCTTTGAGCCGGGGAATTTGGGCGTTTCGCTTGGCCACCATGTGGGATAGCGAATAGCAAATTTTAATTGCTGCACAGGCAGGTTCCTGGTTTGCTAATTATTCGACACGAAAATACCTTTCAATGCAGGGAGAGTATATTAAAAGGGAAATCAATGGCTCTTACAGATAGATAGATACTAAGTTGAAAGTGTAATATAATAAAATTGTATACATTCTTAGTCAGTaattatactgaaatctttaaagggtatccgcactttcatctcctgcctagatttcccttgccatttgagcctttttacaacttggccagcaacagccttcgttggctgacttgcttttgttgtcttttatgggatttcaacacgttaaggtgtaaagaacaacgtaattaaaatgcaagccgtcgtttcttaagctcggatagatgggttcccgactgcgtcatgcgtttaggtgcggtctagtttgttttccattgtttcccgtttcccgtgctgtgggttttgcagtcgcccacgctccgaaattagaaacaagagtcagttgcgggcagcccctccactgatttcagctggggcgccccttcaattcaccggctattatcttaattttaggcacccaaggccccgaggtaagccacaaaactcgacacgtgtttctgggctcgtcctgatgtggtaattgttaattaaaaacactctcgggtcacgttcagtgtgttttaggtgccctattaaagtcataaaaaaaaagctaggcaatccaaccatcgctgggttcgactcttttattggacgtcgttaaggggttttgaaaacaagtttatgttgaaccataactccacatacatataatgctgtctatgaaattggacttgtaatacatatttaatgatacctctgaaggatttatgcgggggaagataagtaattgctttgtttatgaaggtagggaaatatttaaaattaggcgaaaatatgaaaaacaaattatttttatattttagcagcaatagtaagatgtatgattaagattataaaaatatttattcaattctaagaataaggagttttaataaaaaatcgcacaaaagcattaatcaattttcatttcagagtagtttattattaatgttttatatttcaaattaattttctttctgtggaaaagtgtaaaaggacacaagggttggattttccactattaacccattagctggttaaggcgcagagtgaccacgtgtctgttttgtgcccgcaggacctgaaatctttgtcaacttttgtaaaattcgtacatcaatcaattggccaagaatcatccatcaggcaatctgagcgctggctaaccgaaaacacaggaagtgcggtgccaaa

## **Legend**

Intergenic region: xxxxxx

Intron: xxxxxx

UTR: XXXXXX

CDS: XXXXXX

Start and/or Stop codon: **XXX**

gRNA PAM: **XXX**

Point mutation(to prevent the donor to be cutted)**: X**

gRNA target sequence: **XXXXXX**

Linker: xxx

GFP: XXXXXX

Arm primers(The border of arm): XXXXXX
