# Supplementary material for: Role of the m6A methyltransferase Mettl16 in Drosophila development
Source: PLoS Genet. 2026 Sep 24;22(9):e1012317. doi: 10.1371/journal.pgen.1012317 (PMC13608003; doi:10.1371/journal.pgen.1012317)
Supplement: Supplementary file 3 [file pgen.1012317.s019.docx]

Dear Dr. Cadigan,

Thank you very much for handling our manuscript. We are very encouraged by the reviewers’ positive assessment. We wish to thank the two reviewers for their constructive and insightful suggestions, which have made our manuscript more rigorous and complete. We have responded to their comments (copied below in *blue italics*) in full, with new experimental results. We have also revised the text extensively and highlighted the changes in blue. Our original blot/gel image data have been included in the Supporting Information. We look forward to your final editorial decision on our work.

Reviewer #1: The authors have presented a well written manuscript that provides convincing evidence that Mettl16 regulates alternative splicing of mRNAs via the U6 snRNA, and that loss of Mettl16 function results in defects in reproductive tissue regeneration, wing function and climbing rate. Overall this is a well presented and argued manuscript but I have a few questions and concerns as outlined below.

We thank the reviewer for the encouraging and positive comments, as well as the constructive suggestions.

The relatively mild LOF phenotype is interesting as splicing of a varied array of target mRNAs was affected by Mettl16 loss, and a heterozygous deficiency that removes all of the U6 snRNAs enhances the Mettl16 phenotype to lethality. This indicates that Mettl16 regulates alternative splicing in varied tissues and that some are more sensitive to its loss. Can the authors explain why the complete loss of Mettl16 alone has such a mild phenotype, or does loss of Mettl16 only partially abrogate U6 snRNA function?

We thank the reviewer for this insightful question. The mild phenotype of *Mettl16* mutants may be because the total U6 level remains unchanged, while only the m^6^A modification of U6 is reduced. We agree with the reviewer that loss of Mettl16 only partially abrogates U6 function. As reported previously, the m^6^A mark at U6 enhances splicing efficiency specifically for a subset of pre-mRNAs, which bear an A at the +4 position of the 5′ splice site and contain exon sequences weakly recognized by U5 snRNA (Ishigami, et al, 2021, PMID: 34050143). Certain tissues, such as the reproductive and nervous systems, might be enriched for m^6^A-sensitive targets, making them more vulnerable to Mettl16 loss.

The synthetic lethality with heterozygous U6 deficiency supports this "partial abrogation" model. When U6 dosage is reduced by 50%, the combined stress of a reduced U6 pool plus loss of m^6^A-mediated splicing fidelity exceeds the functional threshold of the spliceosome, resulting in lethality. We have incorporated these points into our Discussion.

The GFP knock-in allele shown via schematic in Figure 2 and in the text on page 13 (it would help if there were page and line numbers) does not have any associated evidence to demonstrate that it is as suggested. It would be beneficial to include the sequence data that confirmed the nature of the allele. The authors use this allele to state that Mettl16 is ubiquitously expressed in the wing disc but it appears to be absent from two rows of cells either side of the dorso-ventral compartment boundary (proneural cells).

We thank the reviewer for this helpful suggestion (page and line numbers were added). The complete sequence information of the *Mettl16-GFP* allele is now provided in Supplementary S1 Text. This file documents the genomic structure of *Mettl16*, the GFP sequence, the gRNA target sequences, and the linker sequence between GFP and the C-terminus of *Mettl16*, together confirming the precise in-frame insertion of the knock-in allele.

We also thank the reviewer for the careful observation regarding the apparent absence of Mettl16-GFP in the two rows of proneural cells at the D-V boundary in Fig. 2B. We noticed that this same region also shows absent DAPI staining (previous Fig. 2B’), suggesting that is likely due to the morphology of the disc cells. We repeated the experiment and took multiple confocal sections through the wing disc. As shown below, Mettl16-GFP appears absent from these two rows in certain confocal sections, but is in fact detectable in other sections. We have therefore replaced the previous panels with new images from better confocal section (new Fig. 2B-B”), demonstrating that Mettl16-GFP is present in these proneural cells.

The authors have conducted a range of experiments to show abnormalities in reproductive organs after loss of Mettl16 and have shown that these defects can be rescued by a genomic construct that contains Mettl16. Given that they have generated two Mettl16 RNAi alleles can they please comment if similar phenotypes can be generated via knockdown. One question that arises from this study is whether there is a greater requirement for Mettl16 in the germline compared to the reproductive somatic tissues and this could be answered via comparison of knockdowns using nosVP16::Gal4 and traffic jam Gal4.

We thank the reviewer for this constructive suggestion. We performed RNAi experiments for both alleles in the male and female reproductive systems. Interestingly, both nanos-Gal4:VP16 and tj-Gal4 drive phenotypes similar to, but weaker than, those of the mutant, and the RNAi phenotype is stronger in males than in females.

When *Mettl16* RNAi was induced by nanos-GAL4:VP16 or tj-GAL4, these females can still lay eggs. However, when we stained these ovaries with DAPI and Orb, they consistently contained egg chambers with abnormal numbers of germline cells and mislocalized Orb staining (Fig. S3B-S3E”).

Male flies in which *Mettl16* RNAi was induced by nanos-GAL4:VP16 or tj-GAL4 produced strongly reduced progeny numbers. Similar to the mutants, their testes showed absence of waste bags and disorganized actin cones (Fig. S3F-S3I”).

Together, the sterility of the *Mettl16* mutants likely arises from combinatorial effects across germline, reproductive somatic tissues, and potentially other organs.

All of the antibodies used are not listed in the material and methods. I would particularly like to know which phospho-histone H3 antibody was used. If it was the antibody that detects Ser10 phosphorylation then it should only detect condensed chromatin during mitosis and meiosis. This does not appear to be the case in Figure S2D and needs to be explained.

We really appreciate the reviewer’s important question. In Fig S2C-D, we actually used a Histone H3 antibody (not a phospho-Histone H3 antibody) to label nuclei from mitosis through meiosis, including the 64-cell round spermatids. Therefore, the staining pattern is expected and does not reflect phosphorylated H3. We have now added all antibody information in the Materials and Methods section. We apologize for the confusion.

Figures S3 and S4 appear to be misnamed (inverted) with respect to the text.

We thank the reviewer for pointing out our mistake and have corrected this accordingly.

The authors indicate that the genomic rescue transgene can restore normal splicing patterns (Figures 8C-D). While this appears to be the case for three of the examples shown in the figure, gprk2 only shows a partial reversion to the wildtype splicing pattern.

We thank the reviewer for this careful observation. Indeed, while the genomic rescue transgene substantially restores normal splicing patterns for the majority of tested targets, *gprk2* exhibits only a partial reversion to the WT splicing pattern (Fig 7C-D, S7C). We have revised the corresponding text to accurately reflect this difference.

In our Western blot analysis, we found that Mettl16 levels in the genomic rescue flies were still lower than those in WT flies (Fig 3G). *gprk2* has thirteen exons and a complex splicing pattern; it is also expressed in diverse tissues including the nervous system, germline, and imaginal discs, and is under circadian clock control. It is likely that *gprk2* requires a full dose of Mettl16 to achieve proper splicing. We have added this information to the text.

On page 20 the authors stated that “…observed nuclear co‑localization of GFP‑Mettl16 and mRFP‑Amus in transfected S2 cells”. While both are in the nucleus there is not enough resolution to state that they co-localise (to the extent of protein-protein interaction).

We agree with the reviewer and have changed the sentence to “In S2 cells, both GFP‑Mettl16 and mRFP‑Amus localize to the nucleus”.

The discussion also states “Drosophila Mettl3 mutants also exhibit abnormal wing posture, reduced locomotor activity, and flightlessness, though the underlying mechanisms remain unclear.” I have no issues with this statement but there are known mutants with similar wing postures (e.g. dpp, how). Can literature descriptions of mutants with similar phenotypes be correlated with any of the known splicing or downstream targets of Mettl3.

We thank the reviewer for this thoughtful suggestion. Indeed, the "held-out wing" phenotype is shared by several *Drosophila* mutants affecting distinct aspects of wing/thorax development, including *dpp* and *how*. We have expanded the discussion to incorporate these literatures. While Mettl3 loss recapitulates aspects of these mutant phenotypes, the underlying molecular mechanisms are not fully resolved. Current evidence suggests the phenotype likely does not result from splicing alteration of a single gene (as *Sxl* does in sex determination), but rather from cumulative effects across many targets in both muscle and neuronal tissues. We have revised the relevant passage as follows:

“*Drosophila* *Mettl3* mutants also exhibit abnormal wing posture, reduced locomotor activity, and flightlessness. The “held-out wing” phenotype is shared by several *Drosophila* mutants affecting distinct aspects of wing/thorax development. For instance, alleles of *dpp* that disrupt the 3' cis-regulatory *dpp^heldout^* enhancer produce held-out wings (St. Johnston et al., 1990; Hepker et al., 1999), while *how* (*held out wings*) encodes a KH-domain RNA-binding protein required in muscle development (Zaffran et al., 1997; Baehrecke, 1997). Recent work shows that Mettl3 is required in both muscle and neurons, possibly through regulation of many different genes (Heemken, LMU Munich 2025). It will be interesting to dissect and compare the mechanisms of Mettl3- and Mettl16-dependent behavior defects in the future.”

Reviewer #2: Song et al. report the characterization of the Drosophila m6A methyltransferase Mettl16.
This is a very interesting work, as Mettl16 is conserved across all domains of life, but has not been studied through development. So far, the role of Mettl16 in Drosophila remains obscure.
The authors show that, different than in mammal systems, mutant Mettl16 are viable. However, Mettl16 mutants show several developmental phenotypes, including a strong delay in pupariation and eclosion, defects in wing position, and the lack of the ability to fly. Moreover, male and female mutant individuals are sterile. The authors also characterize the defects these mutants have during oogenesis and spermatogenesis. They describe small ovaries and that males are defective in spermatid elongation and individualization. It is also demonstrated that Mettl16 interacts with U6 snRNA of the splicesome complex and is required for m6A editing.

This work is a complete characterization of Mettl16 through development and also describe some of its specific RNA targets. Overall, it is well written and advances the knowledge on one of the key processes of RNA metabolism controlling gene expression, i.e., the chemical modifications of RNA, such as addition of N6-methyladenosine.

We thank the reviewer for the encouraging and positive comments, as well as the insightful suggestion.

Minor points.
Fig. 5. To me, it is not clear that the O-propargyl-puromycin (OPP) incorporation assay in the wing imaginal disc is the right assay to proof a role in mRNA translation. Can the authors explain why did they chose this assay? Firstly, the activation of the TOR pathway not necessarily triggers protein synthesis. Nevertheless, the assay overexpressing Rheb lacks a control showing that indeed TOR is being activated in the posterior compartment of the imaginal disc.

We thank the reviewer for this insightful comment. A similar puromycin-based assay, Surface Sensing of Translation (SUnSET) was used in HEK293T cells to show reduced signal upon METTL16 knockout (Su, et al, 2022, PMID: 35145225). We therefore sought to test whether Mettl16 plays a similar role in *Drosophila* tissues using the OPP assay, which is widely used for ribosomal protein mutants (Kiparaki and Baker, 2023, PMID: 37862174).

We used Rheb primarily following a paper from J. P. Vincent’s group, who used Rheb overexpression and Rheb RNAi as positive and negative controls for OPP staining (Recasens-Alvarez, et al, 2021, PMID: 33495632). Other groups have also used *Tor* mutants or the repressive 4E-BP^TA^ to manipulate the TOR pathway in their OPP assays (Lee, et al, 2018, PMID: 30253171; Baumgartner, et al, 2021, PMID: 33495633). It has been reported that none of the commercial phospho-S6K antibodies work in immunostaining; A. A. Teleman’s group therefore generated an antibody that detects phosphorylation of the S6K target, RpS6. Unfortunately, this antibody is not available, but their paper has already shown that overexpression of Rheb in the posterior compartment leads to strong activation of pRpS6 staining (Romero-Pozuelo, et al, 2017, PMID: 28829945).

Furthermore, in our revised version, we added a negative control through a different pathway for our OPP assay. PPP1R15 (also known as GADD34) is a phosphatase in the integrative stress response (ISR), and its RNAi leads to enhanced phosphorylation of eIF2α (Langton, et al, 2021, PMID: 34914692), which suppresses global translation as indicated by reduced OPP staining in the posterior compartment (Fig. S4C-C”).

Second, to show a clear role on "de novo" synthesis of proteins, they should perform ex vivo experiments of radioactive methionine incorporation in any tissue, such as imaginal discs or testes. Otherwise, they could make in vitro translation assays adding recombinant Mettl16 and or depleting it from the extract. Also, can the authors perform an IP or pull down and see that no translation factor is co-immunoprecipitated?
Otherwise, the possible involvement of Mettl16 in mRNA translation can be completely omitted and would not affect the global message of the paper.

We thank the reviewer for the suggested experiments. Indeed, methionine incorporation is a gold-standard assay for “de novo” protein synthesis. Unfortunately, we do not have access to radioactive facilities. We are currently trying to develop non-radioactive methods using methionine analogs such as HPG (L-homopropargylglycine) and AHA (L-azidohomoalanine) based assays, which are not yet well-established for *Drosophila* imaginal discs. We will also attempt to perform in vitro translation assays using recombinant Mettl16 in the future. In addition, we did not detect strongly enriched translation factors in our IP-mass spec experiments.

Overall, we agree with the reviewer that our translation analysis is preliminary. Since it still provides some useful information, we have moved it to Supplementary Fig. S4.
