# Supplementary material for: Role of the m6A methyltransferase Mettl16 in Drosophila development
Source: PLoS Genet. 2026 Sep 24;22(9):e1012317. doi: 10.1371/journal.pgen.1012317 (PMC13608003; doi:10.1371/journal.pgen.1012317)
Supplement: Peer review history

# Peer review: Decision Letter 0

**DOI:** 10.1371/journal.pgen.1012317.r001

**Authors:** Ken Cadigan, Giovanni Bosco

3 Jul 2026

PGENETICS-D-26-00483

Role of the m6A methyltransferase Mettl16 in Drosophila development

PLOS Genetics

Dear Dr. Yan,

Thank you for submitting your manuscript to PLOS Genetics. After careful consideration, we feel that it has merit but does not fully meet PLOS Genetics’s publication criteria as it currently stands. Therefore, we invite you to submit a revised version of the manuscript that addresses the points raised during the review process.

Please submit your revised manuscript within by Aug 02 2026 11:59PM. If you will need more time than this to complete your revisions, please reply to this message or contact the journal office at plosgenetics@plos.org. When you’re ready to submit your revision, log on to https://www.editorialmanager.com/pgenetics/ and select the ‘Submissions Needing Revision’ folder to locate your manuscript file.

Please include the following items when submitting your revised manuscript:

* A letter that responds to each point raised by the editor and reviewer(s). You should upload this letter as a separate file labeled ‘Response to Reviewers’. This file does not need to include responses to formatting updates and technical items listed in the 'Journal Requirements' section below.

* A marked-up copy of your manuscript that highlights changes made to the original version. You should upload this as a separate file labeled 'Revised Manuscript with Track Changes'.

* An unmarked version of your revised paper without tracked changes. You should upload this as a separate file labeled 'Manuscript'.

If you would like to make changes to your financial disclosure, competing interests statement, or data availability statement, please make these updates within the submission form at the time of resubmission. Guidelines for resubmitting your figure files are available below the reviewer comments at the end of this letter.

As the corresponding author, your ORCID iD is verified in the submission system and will appear in the published article. PLOS supports the use of ORCID, and we encourage all coauthors to register for an ORCID iD and use it as well. Please encourage your coauthors to verify their ORCID iD within the submission system before final acceptance, as unverified ORCID iDs will not appear in the published article. Only the individual author can complete the verification step; PLOS staff cannot verify ORCID iDs on behalf of authors.

We look forward to receiving your revised manuscript.

Kind regards,

Ken M. Cadigan, PhD

Academic Editor

PLOS Genetics

Giovanni Bosco

Section Editor

PLOS Genetics

Aimée Dudley

Editor-in-Chief

PLOS Genetics

Anne Goriely

Editor-in-Chief

PLOS Genetics

**Additional Editor Comments (if provided):**

**Journal Requirements:**

If the reviewer comments include a recommendation to cite specific previously published works, please review and evaluate these publications to determine whether they are relevant and should be cited. There is no requirement to cite these works unless the editor has indicated otherwise.

1) Please upload all main figures as separate Figure files in .tif or .eps format. For more information about how to convert and format your figure files please see our guidelines:

https://journals.plos.org/plosgenetics/s/figures

2) Please amend your detailed Financial Disclosure statement. This is published with the article. It must therefore be completed in full sentences and contain the exact wording you wish to be published.

i) State what role the funders took in the study. If the funders had no role in your study, please state: "The funders had no role in study design, data collection and analysis, decision to publish, or preparation of the manuscript."

ii) If any authors received a salary from any of your funders, please state which authors and which funders..

If you did not receive any funding for this study, please simply state: u201cThe authors received no specific funding for this work.u201d.

3)Thank you for submitting your data to a repository. We notice that while accessing the data using the accession number you have provided (GSE324097) is still private. Your manuscript has now been provisionally accepted, so at this time, we ask that you please make sure that the data is publicly available. Kindly provide a revised Data Availability Statement including the information.

4)PLOS now requires that authors provide the original uncropped and unadjusted images underlying all blot or gel results reported in a submission’s figures or Supporting Information files. This policy and the journal’s other requirements for blot/gel reporting and figure preparation are described in detail at https://journals.plos.org/plosone/s/figures#loc-blot-and-gel-reporting-requirements and https://journals.plos.org/plosone/s/figures#loc-preparing-figures-from-image-files.

When you submit your revised manuscript, please ensure that your figures adhere fully to these guidelines and provide the original underlying images for all blot or gel data reported in your submission. See the following link for instructions on providing the original image data: https://journals.plos.org/plosone/s/figures#loc-original-images-for-blots-and-gels.

In your cover letter, please note whether your blot/gel image data are in Supporting Information or posted at a public data repository, provide the repository URL if relevant, and provide specific details as to which raw blot/gel images, if any, are not available. Email us at customercare@plos.org if you have any questions.

**Reviewers' comments:**

Reviewer's Responses to Questions

**Comments to the Authors:**

**Please note here if the review is uploaded as an attachment.**

Reviewer #1: The authors have presented a well written manuscript that provides convincing evidence that Mettl16 regulates alternative splicing of mRNAs via the U6 snRNA, and that loss of Mettl16 function results in defects in reproductive tissue regeneration, wing function and climbing rate. Overall this is a well presented and argued manuscript but I have a few questions and concerns as outlined below.

The relatively mild LOF phenotype is interesting as splicing of a varied array of target mRNAs was affected by Mettl16 loss, and a heterozygous deficiency that removes all of the U6 snRNAs enhances the Mettl16 phenotype to lethality. This indicates that Mettl16 regulates alternative splicing in varied tissues and that some are more sensitive to its loss. Can the authors explain why the complete loss of Mettl16 alone has such a mild phenotype, or does loss of Mettl16 only partially abrogate U6 snRNA function?

The GFP knock-in allele shown via schematic in Figure 2 and in the text on page 13 (it would help if there were page and line numbers) does not have any associated evidence to demonstrate that it is as suggested. It would be beneficial to include the sequence data that confirmed the nature of the allele. The authors use this allele to state that Mettl16 is ubiquitously expressed in the wing disc but it appears to be absent from two rows of cells either side of the dorso-ventral compartment boundary (proneural cells).

The authors have conducted a range of experiments to show abnormalities in reproductive organs after loss of Mettl16 and have shown that these defects can be rescued by a genomic construct that contains Mettl16. Given that they have generated two Mettl16 RNAi alleles can they please comment if similar phenotypes can be generated via knockdown. One question that arises from this study is whether there is a greater requirement for Mettl16 in the germline compared to the reproductive somatic tissues and this could be answered via comparison of knockdowns using nosVP16::Gal4 and traffic jam Gal4.

All of the antibodies used are not listed in the material and methods. I would particularly like to know which phospho-histone H3 antibody was used. If it was the antibody that detects Ser10 phosphorylation then it should only detect condensed chromatin during mitosis and meiosis. This does not appear to be the case in Figure S2D and needs to be explained.

Figures S3 and S4 appear to be misnamed (inverted) with respect to the text.

The authors indicate that the genomic rescue transgene can restore normal splicing patterns (Figures 8C-D). While this appears to be the case for three of the examples shown in the figure, gprk2 only shows a partial reversion to the wildtype splicing pattern.

On page 20 the authors stated that “…observed nuclear co‑localization of GFP‑Mettl16 and mRFP‑Amus in transfected S2 cells”. While both are in the nucleus there is not enough resolution to state that they co-localise (to the extent of protein-protein interaction).

The discussion also states “Drosophila Mettl3 mutants also exhibit abnormal wing posture, reduced locomotor activity, and flightlessness, though the underlying mechanisms remain unclear.” I have no issues with this statement but there are known mutants with similar wing postures (e.g. dpp, how). Can literature descriptions of mutants with similar phenotypes be correlated with any of the known splicing or downstream targets of Mettl3.

Reviewer #2: Song et al. report the characterization of the Drosophila m6A methyltransferase Mettl16.

This is a very interesting work, as Mettl16 is conserved across all domains of life, but has not been studied through development. So far, the role of Mettl16 in Drosophila remains obscure.

The authors show that, different than in mammal systems, mutant Mettl16 are viable. However, Mettl16 mutants show several developmental phenotypes, including a strong delay in pupariation and eclosion, defects in wing position, and the lack of the ability to fly. Moreover, male and female mutant individuals are sterile. The authors also characterize the defects these mutants have during oogenesis and spermatogenesis. They describe small ovaries and that males are defective in spermatid elongation and individualization. It is also demonstrated that Mettl16 interacts with U6 snRNA of the splicesome complex and is required for m6A editing.

This work is a complete characterization of Mettl16 through development and also describe some of its specific RNA targets. Overall, it is well written and advances the knowledge on one of the key processes of RNA metabolism controlling gene expression, i.e., the chemical modifications of RNA, such as addition of N6-methyladenosine.

Minor points.

Fig. 5. To me, it is not clear that the O-propargyl-puromycin (OPP) incorporation assay in the wing imaginal disc is the right assay to proof a role in mRNA translation. Can the authors explain why did they chose this assay? Firstly, the activation of the TOR pathway not necessarily triggers protein synthesis. Nevertheless, the assay overexpressing Rheb lacks a control showing that indeed TOR is being activated in the posterior compartment of the imaginal disc. Second, to show a clear role on "de novo" synthesis of proteins, they should perform ex vivo experiments of radioactive methionine incorporation in any tissue, such as imaginal discs or testes. Otherwise, they could make in vitro translation assays adding recombinant Mettl16 and or depleting it from the extract. Also, can the authors perform an IP or pull down and see that no translation factor is co-immunoprecipitated?

Otherwise, the possible involvement of Mettl16 in mRNA translation can be completely omitted and would not affect the global message of the paper.

**********

**Have all data underlying the figures and results presented in the manuscript been provided?**

Large-scale datasets should be made available via a public repository as described in the *PLOS Genetics*  data availability policy, and numerical data that underlies graphs or summary statistics should be provided in spreadsheet form as supporting information.

Reviewer #1: Yes

Reviewer #2: Yes

**********

PLOS authors have the option to publish the peer review history of their article (what does this mean?). If published, this will include your full peer review and any attached files.

If you choose “no”, your identity will remain anonymous but your review may still be made public.

**Do you want your identity to be public for this peer review?**  For information about this choice, including consent withdrawal, please see our Privacy Policy.

Reviewer #1: No

Reviewer #2: **Yes:**  Greco Hernández

[NOTE: If reviewer comments were submitted as an attachment file, they will be attached to this email and accessible via the submission site. Please log into your account, locate the manuscript record, and check for the action link "View Attachments". If this link does not appear, there are no attachment files.]

**Figure resubmission:**

**Reproducibility:**

To enhance the reproducibility of your results, we recommend that authors deposit laboratory protocols in protocols.io, where a protocol can be assigned its own identifier (DOI) such that it can be cited independently in the future. Additionally, PLOS ONE offers an option to publish peer-reviewed clinical study protocols. Read more information on sharing protocols at https://plos.org/protocols?utm_medium=editorial-email&utm_source=authorletters&utm_campaign=protocols

# Author comment: Author response to Decision Letter 1

**DOI:** 10.1371/journal.pgen.1012317.r002

12 Aug 2026

Attachment Submitted filename: Response to Reviewers.docx

# Editor report: Decision Letter 1

**DOI:** 10.1371/journal.pgen.1012317.r003

**Authors:** Ken Cadigan, Giovanni Bosco

11 Sep 2026

Dear Dr Yan,

We are pleased to inform you that your manuscript entitled "Role of the m6A methyltransferase Mettl16 in Drosophila development" has been editorially accepted for publication in PLOS Genetics. Congratulations!

Before your submission can be formally accepted and sent to production you will need to complete our formatting changes, which you will receive in a follow up email. Please be aware that it may take several days for you to receive this email; during this time no action is required by you. Please note: the accept date on your published article will reflect the date of this provisional acceptance, but your manuscript will not be scheduled for publication until the required changes have been made.

Once your paper is formally accepted, an uncorrected proof of your manuscript will be published online ahead of the final version, unless you’ve already opted out via the online submission form. If, for any reason, you do not want an earlier version of your manuscript published online or are unsure if you have already indicated as such, please let the journal staff know immediately at plosgenetics@plos.org.

In the meantime, please log into Editorial Manager at https://www.editorialmanager.com/pgenetics/, click the "Update My Information" link at the top of the page, and update your user information to ensure an efficient production and billing process. Note that PLOS requires an ORCID iD for all corresponding authors. Therefore, please ensure that you have an ORCID iD and that it is validated in Editorial Manager. To do this, go to ‘Update my Information’ (in the upper left-hand corner of the main menu), and click on the Fetch/Validate link next to the ORCID field.  This will take you to the ORCID site and allow you to create a new iD or authenticate a pre-existing iD in Editorial Manager.

If you have a press-related query, or would like to know about making your underlying data available (as you will be aware, this is required for publication), please see the end of this email. If your institution or institutions have a press office, please notify them about your upcoming article at this point, to enable them to help maximise its impact. Inform journal staff as soon as possible if you are preparing a press release for your article and need a publication date.

Thank you again for supporting open-access publishing; we are looking forward to publishing your work in PLOS Genetics!

Yours sincerely,

Ken M. Cadigan

Academic Editor

PLOS Genetics

Giovanni Bosco

Section Editor

PLOS Genetics

Aimée Dudley

Editor-in-Chief

PLOS Genetics

Anne Goriely

Editor-in-Chief

PLOS Genetics

www.plosgenetics.org

BlueSky: @plos.bsky.social

----------------------------------------------------

Comments from the reviewers (if applicable):

To expedite the handling of the manuscript, I carefully examined the reviewers' critiques and your responses and revisions. I find that you address all of reviewer 1's concerns in a comprehensive manner. For reviewer 2, you

were not able to measure translation rates with the "gold standard" radioactive incorporation, or other suggested experiments (co-IP; in vitro translation). But I think your moving these results to the supplemental are in line

with the reviewer's suggestion. Therefore, I am happy to accept your manuscript for publication in PLOS Genetics.

----------------------------------------------------

**Data Deposition**

If you have submitted a Research Article or Front Matter that has associated data that are not suitable for deposition in a subject-specific public repository (such as GenBank or ArrayExpress), one way to make that data available is to deposit it in the Dryad Digital Repository. As you may recall, we ask all authors to agree to make data available; this is one way to achieve that. A full list of recommended repositories can be found on our website.

The following link will take you to the Dryad record for your article, so you won't have to re‐enter its bibliographic information, and can upload your files directly:

http://datadryad.org/submit?journalID=pgenetics&manu=PGENETICS-D-26-00483R1

More information about depositing data in Dryad is available at http://www.datadryad.org/depositing. If you experience any difficulties in submitting your data, please contact help@datadryad.org for support.

Additionally, please be aware that our data availability policy requires that all numerical data underlying display items are included with the submission, and you will need to provide this before we can formally accept your manuscript, if not already present.

----------------------------------------------------

**Press Queries**

If you or your institution will be preparing press materials for this manuscript, or if you need to know your paper's publication date for media purposes, please inform the journal staff as soon as possible so that your submission can be scheduled accordingly. Your manuscript will remain under a strict press embargo until the publication date and time. This means an early version of your manuscript will not be published ahead of your final version. PLOS Genetics may also choose to issue a press release for your article. If there's anything the journal should know or you'd like more information, please get in touch via plosgenetics@plos.org.

# Editor report: Acceptance letter

**DOI:** 10.1371/journal.pgen.1012317.r004

**Authors:** Ken Cadigan, Giovanni Bosco

PGENETICS-D-26-00483R1

Role of the m6A methyltransferase Mettl16 in Drosophila development

Dear Dr Yan,

We are pleased to inform you that your manuscript entitled "Role of the m6A methyltransferase Mettl16 in Drosophila development" has been formally accepted for publication in PLOS Genetics! Your manuscript is now with our production department and you will be notified of the publication date in due course.

The corresponding author will soon be receiving a typeset proof for review, to ensure errors have not been introduced during production. Please review the PDF proof of your manuscript carefully, as this is the last chance to correct any errors. Please note that major changes, or those which affect the scientific understanding of the work, will likely cause delays to the publication date of your manuscript.

Soon after your final files are uploaded, unless you have opted out or your manuscript is a front-matter piece, the early version of your manuscript will be published online. The date of the early version will be your article's publication date. The final article will be published to the same URL, and all versions of the paper will be accessible to readers.

For Research Articles, you will receive an invoice from PLOS for your publication fee after your manuscript has reached the completed accept phase. If you receive an email requesting payment before acceptance or for any other service, this may be a phishing scheme. Learn how to identify phishing emails and protect your accounts at https://explore.plos.org/phishing.

Thank you again for supporting PLOS Genetics and open-access publishing. We are looking forward to publishing your work!

With kind regards,

Sharmila Kamatchi

PLOS Genetics

On behalf of:

The PLOS Genetics Team

Carlyle House, Carlyle Road, Cambridge CB4 3DN | United Kingdom

plosgenetics@plos.org | +44 (0) 1223-442823

plosgenetics.org | Twitter: @PLOSGenetics
